# Essential Medicines in Universal Health Coverage: A Scoping Review of Public Health Law Interventions and How They Are Measured in Five Middle-Income Countries

**DOI:** 10.3390/ijerph17249524

**Published:** 2020-12-18

**Authors:** Katrina Perehudoff, Ivan Demchenko, Nikita V. Alexandrov, David Brutsaert, Angela Ackon, Carlos E. Durán, Faris El-Dahiyat, Firdaus Hafidz, Rezwan Haque, Rabia Hussain, Roderick Salenga, Fatima Suleman, Zaheer-Ud-Din Babar

**Affiliations:** 1 Law Center for Health and Life, University of Amsterdam, 1018 WV Amsterdam, The Netherlands; 2 Department of Public Health & Primary Care, Ghent University, 9000 Gent, Belgium; David.Brutsaert@ugent.be; 3 WHO Collaborating Centre for Governance, Accountability, and Transparency in the Pharmaceutical Sector, University of Toronto, 144 College Street, Toronto, ON M5S 3M2, Canada; 4 Forensic Medicine and Medical Law Department, National Medical University ‘O.O. Bogomolec’, 01601 Kyiv, Ukraine; demchenko.ivan@gmail.com; 5 Global Health Law Groningen Research Centre, Department of Transboundary Legal Studies, Faculty of Law, University of Groningen, 9700 AS Groningen, The Netherlands; nikita.v.alexandrov@gmail.com; 6 Directorate of Pharmacy, Ministry of Health, P. O. Box M 44 Accra, Ghana; angiackon@yahoo.com; 7 Clinical Pharmacology Research Group, Department of Basic & Applied Medical Sciences, Ghent University, 9000 Ghent, Belgium; carlos.duran@ugent.be; 8 College of Pharmacy, Al Ain University, 64141 Al Ain, UAE; faris.dahiyat@aau.ac.ae; 9 Department of Health Policy & Management, Universitas Gadjah Mada, Yogyakarta 55281, Indonesia; hafidz.firdaus@ugm.ac.id; 10 Access to Information (a2i) Programme (Former Project Director, SWASTI), Dhaka 1207, Bangladesh; rezwan.haque@outlook.com; 11 Department of Pharmacy (Adjunct), Ranada Prasad Shaha University, Narayanganj 1400, Bangladesh; 12 Faculty of Pharmacy, The University of Lahore, Lahore 54590, Pakistan; rabia.hussain2010@gmail.com; 13 Commonwealth Pharmacists Association, London E1W 1AW, UK; 14 College of Pharmacy, University of the Philippines Manila, Metro Manila 1000, Philippines; rlsalenga@up.edu.ph; 15 Discipline of Pharmaceutical Sciences, University of KwaZulu-Natal, Durban 4041, South Africa; sulemanf@ukzn.ac.za; 16 Department of Pharmacy, University of Huddersfield, Queensgate, Huddersfield HD1 3DH, UK; Z.Babar@hud.ac.uk

**Keywords:** universal health coverage, universal health insurance, health insurance, access to medicines, legislation, drug, insurance, pharmaceutical services, health services accessibility, pharmaceutical policy, middle income country, essential medicines

## Abstract

Very few studies exist of legal interventions (national laws) for essential medicines as part of universal health coverage in middle-income countries, or how the effect of these laws is measured. This study aims to critically assess whether laws related to universal health coverage use five objectives of public health law to promote medicines affordability and financing, and to understand how access to medicines achieved through these laws is measured. This comparative case study of five middle-income countries (Ecuador, Ghana, Philippines, South Africa, Ukraine) uses a public health law framework to guide the content analysis of national laws and the scoping review of empirical evidence for measuring access to medicines. Sixty laws were included. All countries write into national law: (a) health equity objectives, (b) remedies for users/patients and sanctions for some stakeholders, (c) economic policies and regulatory objectives for financing (except South Africa), pricing, and benefits selection (except South Africa), (d) information dissemination objectives (ex. for medicines prices (except Ghana)), and (e) public health infrastructure. The 17 studies included in the scoping review evaluate laws with economic policy and regulatory objectives (*n* = 14 articles), health equity (*n* = 10), information dissemination (*n* = 3), infrastructure (*n* = 2), and sanctions (*n* = 1) (not mutually exclusive). Cross-sectional descriptive designs (*n* = 8 articles) and time series analyses (*n* = 5) were the most frequent designs. Change in patients’ spending on medicines was the most frequent outcome measure (*n* = 5). Although legal interventions for pharmaceuticals in middle-income countries commonly use all objectives of public health law, the intended and unintended effects of economic policies and regulation are most frequently investigated.

## 1. Introduction

Universal access to essential medicines is an integral part of the Sustainable Development Goal (SDG) 3 for health and SDG Target 3.8 on universal health coverage (UHC). Paying out of pocket for medicines imposes a significant burden on people in low- and middle-income countries (LMICs), where medicines account for up to 67% of all public and private health spending [1,2]. In these settings 50–90% of medicines expenses are paid out-of-pocket due to inadequate financial coverage for health services or UHC [1,2]. UHC is defined by the World Health Organization (WHO) as “[E]nsuring that all people have access to needed health services (including prevention, promotion, treatment, rehabilitation and palliation) of sufficient quality to be effective while also ensuring that the use of these services does not expose the user to financial hardship” [1].

It is also stated that “UHC can be accomplished only through the law” [3]. National legislation for UHC establishes rules about the health system’s structure and function in order to provide access to needed health services to the population while ensuring equity and financial coverage for the most vulnerable [4]. Strengthening national legal frameworks to support access to health services has recently featured prominently in global health debates [5,6,7,8]. The 2019 Lancet-O’Neill Institute Commission on the Global Health and Law proposed three legal determinants of health [5]. One of those determinants requires health laws to fulfil each element of UHC: (a) population coverage, (b) inclusion of services, and (c) cost coverage [5].

UHC, and in particular the establishment of legal rules for the population, services, and cost coverage elements of UHC, offers an opportunity to better regulate medicines for assured quality, lower prices, and consistent availability, while promoting equitable service coverage [9]. To this end we propose that five objectives of public health law, if properly embedded in law and implemented in practice, can help attain universal access to essential medicines. The objectives of public health law include: establishing public health infrastructure, applying economic policies (incentives and disincentives) and regulation, transparency and information dissemination, promoting health equity, remedies and sanctions [10]. These objectives offer different strategies in lawmakers’ toolboxes. For example, legal interventions for medicines price control are ineffective if not properly resourced, monitored, and enforced [11,12]. However, to our knowledge no study has investigated how national UHC law employs these different objectives (separately or in concert) in relation to medicines affordability and financing.

The intended and unintended effects of national UHC law on universal access to essential medicines are a point of ongoing debate. Evidence shows that the introduction of health insurance systems can improve access to pharmaceuticals and outcomes in LMIC [9,13]. A review of medicines management in health insurance in LMICs found that studies tended to focus on strategies to influence prescribing, with little attention to strategies for medicines selection, procurement or use [9]. A pre-/post-intervention study suggests Chile’s universal system of health guarantees contributed to better treatment of acute myocardial infarction (with pharmaceuticals, among other treatments) in public hospitals and 1-year survival [13]. An interrupted time-series analysis of Thailand’s Universal Coverage Scheme that expanded medicines coverage to the entire population resulted in increased sales of essential and non-essential medicines for out-patient treatment of non-communicable diseases [14]. The latter study illustrates the potential for UHC-related laws to have unintended effects on medicines prescribing, purchasing, or use. One prominent example of this is the former 15% mark-up on medicines sales allowed at public hospitals in China [15]. This mark-up yielded important revenue for public hospitals providing services for the Urban Employee Basic Medical Insurance scheme [15]. However, the mark-up policy also incentivised over-prescribing, particularly of antibiotics, injections, and hormones [15,16].

Several key gaps in the literature impair a thorough understanding of the full range of effects of national law on access to medicines in middle-income countries. First, there is a scarcity of data on UHC-related laws as the intervention under study. Several reviews examine the role of laws, but also “rules, financial and administrative orders made by governments, non-government organizations [NGOs] or private insurers” on access to medicines [17]. The results in these reviews do not distinguish between the different types of legal instruments used while they have important distinctions for lawmakers and health systems. Legislation that must be adopted by elected lawmakers requires great effort and time to negotiate and approve. The resulting law is usually more static (i.e., remains legally binding throughout political and other changes unless it is repealed or reformed) than lower-level orders (ex. those adopted by the executive branch such as the Ministry of Health), policies, or rules that can be more easily revised or repealed by a government administration. In addition, previous studies group together legal and guidance documents that are made by governments, NGOs, and private insurers, while it is likely that only some of these are legally binding. Whether or not a document is legally binding can affect how well it is implemented, monitored, and enforced in practice.

Second, there is little exploration of which measures and data sources in LMICs are capable of and useful to assess the intentional and unintentional effects of law on access to medicines. One review uses an open definition of outcome measures, while another defines outcome measures as only relating to medicines use, healthcare utilisation, health outcomes, or costs [17,18].

Closely linked to the first two gaps, the third gap is the lack of knowledge about how the objectives in public health law have an impact on traditional (i.e., those above) and other outcome measures of access to medicines. This is a key challenge that lawmakers face. Lawmakers would benefit from more evidence about how the objectives in their public health law toolkits have intentional and unintentional impacts on medicines in the health system.

Fourth, the above gaps in knowledge persist in LMIC settings. Some review papers include data from LMICs, while a minority are exclusively focused on LMICs [17,18]. The scarcity of published evidence from LMICs is corroborated by several reviews [9,18,19].

This study aims to critically assess how UHC-related laws use the objectives of public health law to promote medicines affordability and financing. This study also aims to describe how access to medicines secured through the national law is measured. We propose that using a public health law framework can offer a more holistic view of possible legal strategies to lawmakers seeking to promote universal access to essential medicines as part of UHC in middle-income countries.

## 2. Materials and Methods

This five-country comparative case study includes a legal content analysis of national law related to UHC and medicines and a scoping review of the evidence for measuring access to medicines secured through the national law.

### 2.1. Country Selection

We selected five middle-income countries (Ecuador, Ghana, the Philippines, South Africa, Ukraine) that are at different stages of introducing a national health insurance system that covers people in vulnerable situations. These countries also represent different WHO regions and legal families [20]. (See Table 1).

### 2.2. Legal Analysis

The legal mapping exercise collected national laws online between May–July 2020 if they concerned any aspect of the pricing, affordability and/or financial coverage of pharmaceuticals for people in vulnerable positions, guided by a previous study by Perehudoff et al. [21]. Laws included all relevant legislative acts and regulations; where relevant, orders, instructions and rules implementing laws were also included in order to keep the study manageable. All documents were included if they related to the any of the five objectives of public health law in the context of medicines pricing and affordability. Laws were excluded if they related to controlled medicines, traditional or alternative medicines, the regulation of health professions, pharmaceutical advertising, medicines quality, safety, and/or efficacy, or the rational use of medicines. Pharmaceutical policies were excluded from the formal analysis because this study aims to capture the seldom-studied legal commitments and obligations embedded in national law.

To validate the selection of laws, two authors collected laws for each country in iterative steps: one author (KP) mapped national laws based on data collected from a previous study [21] while a second author (AA, CED, ID, RS, FS), an expert in the national context, systematically verified (for relevance) and retrieved or confirmed laws in their original language. Laws that were identified in the scoping review were considered for inclusion if they met the above criteria.

Authors used national databases to identify laws currently in force (see complete list in Supplementary Material S1). The laws were retrieved in their original language (English, Spanish, Ukrainian). When laws were not published in English, co-authors (CED, ID) translated relevant legal provisions from Spanish and Ukrainian to English. The analysts (KP, ID) scanned and confirmed concepts, definitions, and context in the English translation and where needed, by consulting an online translation from the original language and in liaison with the native speaking co-authors who confirmed or corrected the translation. English-language translations of domestic constitutions were sourced from the Constitute Project (constituteproject.org). See Supplementary Material S4 for the list of national laws included in this study.

Two authors trained in law (KP, ID) established definitions of the objectives of public health law (called “categories” in Table 2, modified from Magnusson et al. [10]), which served as the analytical framework. Authors (KP, ID) categorised the laws in three phases (1-laws from Ukraine to develop and trial the categories and definitions in the framework; 2-laws from Ecuador to refine the framework; 3-laws from remaining three countries) using NVivo 12. Between each phase the coders discussed coding differences and revised the coding definitions. The final framework was organised into five main categories (Infrastructure, Economic policies and regulation, Information, Health equity, and Remedies & sanctions).

### 2.3. Scoping Review

The search was conducted by three authors (NVA, DB, KP) based on peer-reviewed and grey literature to identify evidence of how access to medicines achieved through the national public health law is measured in our five study countries. This structured search was informed by the PRISMA-Sr guidelines [25]. The protocol search and selection strategy was piloted by two authors (KP, NVA), revised, and finalised in May 2020.

Four bibliographic databases (Ovid Medline (1946–Present), Embase (1947–Present), Scopus, and Web of Science) were searched. The search strategies were informed by a University of Toronto librarian and further refined through piloting. The final search strategies for each database are available in Supplementary Material S1. The last search was run on 15 May 2020.

The grey literature search included: (a) a structured Google search in English, Ukranian and Spanish using the search terms “pharmaceutical, medicine, law + respective country”, “лікарські засоби, громадське здоров’я, вплив [Medicines, public health impact, Ukraine]”, “Medicamentos + Ley + Impacto + Ecuador [Medicines + Law + Impact + Ecuador]”, and ““Política farmacéutica” + Ecuador [“Pharmaceutical Policy” + Ecuador]”; (b) a snowball search of the text and references of relevant review articles [9,17,18,19,26,27,28,29,30,31,32,33,34,35,36,37]; (c) a snowball search of the text and references of relevant WHO technical guidance documents [38,39]; and (d) crowdsourcing known publications from among the authors.

The scoping review was designed using a Population-Concept-Context approach. The Population is the five middle-income countries (Ecuador, Ghana, the Philippines, South Africa, Ukraine). The database search included 10 countries: the five study countries plus Bangladesh, Colombia, Indonesia, Jordan, and Turkey, which were excluded from this study at the full-text stage due to insufficient access to primary data sources (national laws for Bangladesh, Colombia, and Turkey) and to legal expertise to assist with the non-English analysis of national law (Indonesia, Jordan). The Concept is essential medicines or pharmaceuticals that are defined by WHO as those used for disease prevention, treatment, and control, and are applicable to most chronic and acute diseases [38,39,40]. The Context is universal health coverage, which is defined in the introduction [20]. Our focus is on UHC schemes that include people in vulnerable situations who are unlikely to be able to afford their medicines.

The inclusion criteria were: (a) study setting in one of the five countries selected; (b) a legal intervention related to or with a conceivable impact on systems-level or patient-level measures of access to medicines; (c) the outcome measures are related to any aspect of access to pharmaceuticals. Eligible articles were not restricted by type of participants in the study countries nor by outcome measure. Excluded articles studied the effects of private or regional/pilot micro insurance schemes that are not governed by national UHC legislation, and analyses that preceded the adoption of a legal intervention of interest (defined in the first part of this study). Review articles were excluded but snowball searched for relevant literature (see grey literature search above).

**Figure 1:**
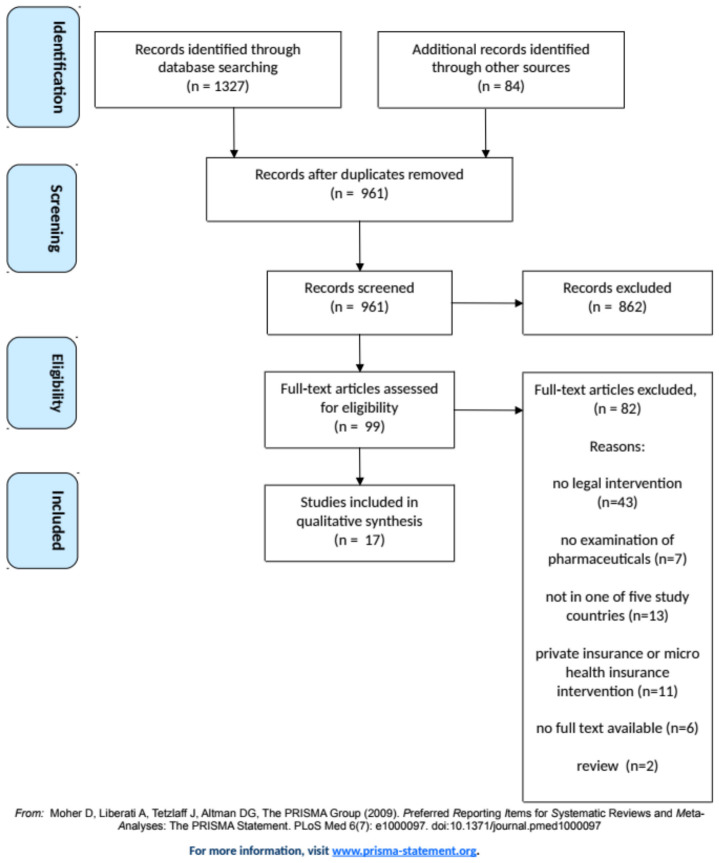
Flow diagram illustrating the selection process of included articles.

English-language database citations were selected in four steps: (a) Automatic and manual removal of duplicates in Mendeley (KP); (b) Title and abstract screening by two blinded reviewers (NVA, DB) in Rayyan for inclusion and exclusion criteria; (c) Blinded full-text screening (NVA, DB) to confirm the eligibility of the study; and (d) Snowball search of the references in eligible studies (NVA, DB, KP). (See Figure 1) Disagreements on eligible articles were resolved through discussion.

One reviewer (NVA) extracted data using a template (see Supplementary Material S4) and the second reviewer (KP, DB, CD) checked the results. No quality/risk of bias assessment was performed on the selected articles. Variables collected from each eligible study included: design, law/legal intervention, public health objectives of the legal intervention, study population (within the five study countries), data source, main outcome measure(s), results. The evidence is presented in a narrative format with the results grouped by the public health objective underlying the legal intervention studied.

## 3. Results

### 3.1. How National Law Uses the Objectives of Public Health Law for Access to Medicines

Sixty laws and regulations were included in this study from five countries (Ecuador *n* = 13; Ghana *n* = 7; Philippines *n* = 10; South Africa *n* = 7; Ukraine *n* = 23). All laws have been adopted except the proposed National Health Insurance Bill in South Africa. All included laws are listed in Supplementary Material S3.

Table 3 shows which objectives of public health law regarding medicines affordability and financing are met in the laws of each country. Laws introducing economic policies and regulations, measures for health equity, and remedies (for users/patients) are identified in each of the five countries. Laws regarding information most often concern health providers’, public authorities’, and economic operators’ responsibility to inform third parties about medicines prices, rather than about pharmaceutical benefits. Sanctions for health providers, public authorities, and economic operators are embedded in four countries.

The content of laws included in this study is summarised below. Where known, non-binding policy or practice is mentioned in square parentheses for topics that were not addressed in law. See the full country summaries in Supplementary Material S4.

#### 3.1.1. Infrastructure

A committee is established to select essential medicines which serves as the pharmaceutical benefits plan (Ecuador, Ukraine). [A National Essential Medicines Committee is prescribed in policy in Ghana, the Philippines and South Africa, rather than in legislation.] In addition, a body regulates medicines prices (Ecuador, Philippines, South Africa, Ukraine). In the Philippines, an independent price negotiation board negotiates with manufacturers on behalf of the DOH and PhilHealth. A committee is established to allocate financial resources and advise on or review the health benefits plan (Ecuador, Ghana, South Africa-proposed) and priority setting for benefits (South Africa-proposed). [In Ghana, operational structures for priority setting are established and in the early stages of implementation.] A centralised public procurement body is established (Ecuador, Ghana, Philippines).

#### 3.1.2. Information

Maximum retail prices of medicines in the private sector must be provided on medicines labels to the public (Ecuador). In the Philippines, manufacturers, importers, distributors, wholesalers, traders, and retailers must display the retail price on medicinal products that may not exceed the maximum retail price. Manufacturers, importers, wholesalers, distributors or pharmacists in South Africa are required by law to inform the public about the single exit price (SEP), availability, applicable pricing system, supply chain, and fees charged by wholesalers, distributors, retailers, and other sellers (for example, as a breakdown on the invoice to the patient). The Director-General may request any information s/he may deem relevant from applicants for a SEP, including any pricing information.

Healthcare providers are obliged to inform patients about the medicines they can receive under the medical guarantee program (Ukraine). [In South Africa health providers must also inform patients about generics, but this duty was not identified in law.] Pharmaceutical workers may not conceal information about lower priced medicines from consumers. (Ukraine)

Public authorities must publish a list of medicines’ prices that has been set (Ecuador); an electronic catalogue of government procurement including prices (Ecuador); the generic names and corresponding brand names of all medicines available on the market, annually in at least two newspapers (Philippines); the trade names of reimbursable medicines, the amount of reimbursement and surcharge (if partially reimbursed), and the retail price per package (Ukraine); the presence of a managed entry agreement, including the INN, trade name, form and dose (Ukraine). Applications for medicines to be considered on the Ukrainian National List of Essential Medicines are published on the Expert Committee’s website after submission.

Members/participants receive information about the benefits package (Ghana, South Africa-proposed legislation), the program of medical guarantees (Ukraine), and the funding of healthcare services (South Africa-proposed).

#### 3.1.3. Economic Policies & Regulation

In terms of public financing, the State is responsible for providing resources to the health sector (Ecuador) and for medicines in the medical guarantee program (Ukraine). Funds from a variety of sources (governmental, individual, employer, other) are pooled to support the social security for peasants and fisherman, the general social security for employees in the formal economy and healthcare (Ecuador), national health insurance (Ghana, Philippines, South Africa-proposed), health care (Ukraine).

The National Essential Medicines List serves as the basis for (Ghana) or the list of pharmaceutical benefits (Ecuador). Health technology assessment has a role in evaluating pharmaceutical benefits (South Africa-proposed). An explicit list of products excluded from a benefits package including (non-exhaustive) products that are not included in the Formulary unless an exception applies (South Africa-proposed), and products that are cost-ineffective ((Philippines, South Africa-proposed). Pharmaceutical benefits must be regularly reviewed (Ecuador, Ghana).

Maximum retail prices apply to essential medicines (Ukraine) and in three scenarios (in Ecuador, related to ‘strategic medicines’, competition failures, and sales above the fixed price and/or emergency conditions). In the Philippines maximum retail prices also apply to medicines for the treatment of chronic and life-threatening conditions, and price negotiation between health insurance purchasers and manufacturers. A single exit price system (ceiling price) is implemented on all medicines registered in South Africa for the private sector, which prohibits bonusing, rebates, or other incentive schemes. This method of transparent pricing establishes appropriate dispensing fees for wholesalers/distributors, pharmacists, and other dispensing health care professionals. A procurement price is established in Ecuador’s public sector. Both countries have an electronic catalogue of government procurement prices.

Anti-competitive practices by dominant firms are prohibited; these include excessive pricing, predatory pricing, margin squeeze, exclusionary acts on certain conditions, refusal to supply scarce goods, buying up scarce supply of intermediate goods needed by a competitor, and refusing to grant a competitor access to an essential facility when feasible to do so (South Africa), and forming a cartel, hoarding products, or profiteering (Philippines). To prevent excessive pricing in an emergency, the prices of basic necessities including pharmaceuticals are automatically frozen at their prevailing price (Philippines). Government use or compulsory licenses may be granted to remedy anticompetitive practices by a patent holder, or in an emergency situation or in the public interest (all five countries). Data exclusivity is granted for five years (Ecuador, Ukraine) and may be extended if certain conditions are met by another six years (Ukraine).

#### 3.1.4. Health Equity

The right to health is recognised in law (Ecuador, Ghana, Philippines, South Africa, Ukraine) and specifically includes free of charge access to medicines (Ecuador), or a right to medical/health care (Ghana, South Africa, Ukraine).

The State must create conditions for an effective medical service accessible to all citizens (Ukraine), to realise health rights within available resources (South Africa), to make essential goods affordable to all (Philippines), and to ensure the availability (Ukraine) and affordability (Ecuador) of essential medicines. The State must guarantee specialised, timely and free-of-charge care to people suffering from catastrophic or highly complex diseases (Ecuador). The President must report on steps taken towards the realisation of the ‘right to good health’ (Ghana).

Medicines in the benefits plan are provided free of charge to all people attending public and out-patient health facilities (Ecuador) to in-patients (Philippines, Ukraine). In Ukraine certain categories of patients also have a right to free medicines, such as children with disabilities living in particular regions and people with disabilities resulting from war, veterans, injured children and their parents. Medicines are provided free of charge or at special prices for out-patients in case of diseases (cardiovascular diseases, type II diabetes, bronchial asthma) (Ukraine).

A variety of exceptions and waivers for UHC contributions are in place to support the financial coverage of vulnerable groups, including but not limited to ‘indigent’ members (Philippines, Ghana), and others (Philippines, South Africa-proposed, Ukraine).

#### 3.1.5. Remedies & Sanctions

A complaints procedure to resolve patients’/members’ grievances in the context of UHC must be established in Ghana, the Philippines, and South Africa. In Ukraine, the State must establish responsibility for rights violations in the field of healthcare, which requires the State and other relevant entities to take measures to restore rights, protect citizens’ legitimate interests, and compensate for damage. Consumer protection and remedies for violating the rights of users are provided in Ecuador’s Constitution. Public service providers and producers of consumer goods are civilly and criminally liable for inadequate provision of services or provision of a poor-quality product. The state can be liable for civil damages due to negligence and carelessness in the provision of or deficiency in the public services under its responsibility.

For health providers and economic operators, a range of penalties and/or sanctions are established in relation to medicines pricing and price control. Penalties and sanctions may be applied for exceeding the fixed price/price ceilings (to pharmaceutical manufacturers and medicines providers in Ecuador; to any manufacturer, importer, trader, distributor, wholesaler, retailer or other entity in the Philippines; anyone who contravenes the provisions for the single exit price in South Africa). In the Philippines government officials and/or employees guilty of conspiring in these acts may also face criminal penalties and/or administrative sanctions. Moreover, in the Philippines a penalty of imprisonment and fines may be applied for manipulating prices by hoarding, profiteering or illegally combining or forming a cartel, and other acts aiming to restrict the trade of medicines. In Ecuador, penalties and sanctions may be applied to pharmaceutical manufacturers and medicines providers for exceeding profit margins established by the national authority. Penalties may be applied to economic operators guilty of anti-competitive behaviour (Ecuador, Philippines, South Africa).

Sanctions and penalties can be given to health providers for failing to provide information about medicines prices and substitution for lower-cost alternatives (established by law). In South Africa pharmacists who fail to inform members of the public at point of dispensing about the benefits of generic substitution, and doing generic substitution unless expressly forbidden to do so or the generic price is higher than brand, or there is no substitute. Any person who contravenes the provisions for generic substitution is liable for a fine or imprisonment. In Ecuador, dispensers (called ‘pharmaceutical establishments’) can be sanctioned for failing to stock generic versions of brand name medicines that appear in the National Table of Basic Medicines. Penalties also apply to pharmacies for failing to provide consumers with access to the retail medicines prices of brand medicines and generic equivalents sold, and for violating the provision in the Generic Medicines Act. In Ukraine, medical and pharmaceutical workers are prohibited from and liable for advertising medicines, and for failing to provide or providing inaccurate information about medicines with the same active substance available at a lower price.

General sanctions for public authorities are embedded in UHC legislation; these may also apply to the affordability and financing of pharmaceutical benefits. Disciplinary proceedings apply to public servants for offences (i.e., improper demand or collection of unauthorised fees, Ghana). Health institutions and health providers are liable for refusing to give benefits to PhilHealth members entitled to them or charging patients for medicines covered by the program (Philippines). Legislation establishes penalties for public servants in the UHC scheme (Ghana); describe corrupt acts in detail in relation to public procurement (civil and administrative penalties in the Philippines); for false information or representation towards the Fund, or use of money from Fund under false pretences (South Africa- proposed); and for people guilty of violating legislation on the State financial guarantees of medical care (Ukraine).

### 3.2. Measuring Access to Medicines Secured through National Law

A total of 17 studies were identified for inclusion in the review. The search of Ovid Medline, Embase, Scopus, and Web of Science databases provided a total of 1327 citations (references). After including articles located from the grey literature and adjusting for duplicates 961 articles remained. Of these, 862 studies were discarded because they did not meet the inclusion criteria and the full text of 99 studies was assessed for eligibility. Of these, 82 articles were excluded because the design did not evaluate a legal intervention (*n* = 43), the study did not specifically examine pharmaceuticals (*n* = 7), the setting was not in one of the five study countries (*n* = 13), the study concerned private insurance or micro health insurance (*n* = 11), no full text was available (*n* = 6), or the articles were reviews or summaries of original research already included in screening (*n* = 2). Seventeen studies were included in the review. Figure 1 illustrates the inclusion process. Table 4 summarises the studies’ designs, legal interventions, study populations and data sources, and main outcome measures and results. Supplementary Material S2 presents the search results.

Study settings were Ghana (*n* studies = 7); Ecuador (*n* = 5); the Philippines and South Africa (*n* = 2 each); and the Ukraine (*n* = 1). All studies were observational. The main study designs were (not mutually exclusive) cross-sectional descriptive (*n* = 8), time series analyses (*n* = 5), convergent parallel mixed methods (*n* = 2), or longitudinal, pre-post-intervention, or cohort (*n* = 1 each).

The main outcome measures were (not mutually exclusive) a change in out-of-pocket spending, or cost of or co-payment for medicines for the patient (*n* = 5), medicines utilisation (*n* = 3), private sector retail price (*n* = 2) or ceiling price (*n* = 2), hospital expenditure on medicines (*n* = 1), medicines availability in mission facilities (*n* = 1), INNs included in the reimbursement system (*n* = 1), medicines sales volumes and market shares (*n* = 1), number of patients covered by reimbursement system (*n* = 1), number of pharmacies enrolled in reimbursement system (*n* = 1), budget allocation by Ministry of Health to pharmaceutical reimbursement (*n* = 1), potential budget impact of court-ordered medicines if the decision was extended to all eligible patients (*n* = 1), and a relationship between the Essential Medicines Committee and the process for expanding the reimbursement list (*n* = 1), and the frequency of successful court claims for access to publicly funded medicines (*n* = 1). Self-reported outcome measures included household knowledge, attitudes, and practices with regards to medicines (price) regulation (*n* = 1); opinions and beliefs about change in demand for and availability of medicines, and awareness of medicines coverage (*n* = 1); and major complaints reported by pharmaceutical sector stakeholders (*n* = 2).

The main legal interventions were concerning framework and implementing legislation for national health insurance (Ghana *n* = 7 studies; Ukraine *n* = 1), of which two studies were related to free maternal health care in Ghana. Other legal interventions related to constitutional rights to access medicines (Ecuador *n* = 3), medicines price regulations (South Africa *n* = 2; Philippines *n* = 2), and a decree allowing hospital drug and therapeutic committees to select and procure drugs non-essential medicines (Ecuador *n* = 1). Studies investigating the effects of legal interventions can be primarily characterised (not mutually exclusive) as having economic policy and regulatory objectives (*n* = 14), health equity objectives (*n* = 10), information dissemination objectives (*n* = 3), infrastructure objectives (*n* = 2), and remedies and sanctions (*n* = 1).

## 4. Discussion

In the five MICs we studied, laws in most countries embed public health infrastructure, such as essential medicines or benefits selection committees; economic policies for and regulation of public financing, pooled financing, medicines pricing, and benefits selection; information dissemination, such as about medicines prices; health equity including rights, obligations, and equitable financing; and remedies for users/patients and sanctions for stakeholders. In the national laws we analysed, economic policy and regulatory objectives frequently appeared together with informational and health equity measures. Most evaluations of legal interventions investigate outcomes related to economic policies and regulations and health equity, most commonly a measure of patients’ out-of-pocket spending on medicines, or medicines cost or patient co-payment for medicines. Few studies evaluated legal interventions for information dissemination (ex. on patient knowledge, attitudes and behaviours towards lower-cost generics), for public health infrastructure (ex. centralised and decentralised medicines selection committees on medicines utilisation), and for remedies or sanctions. This study offers an overview for law makers in middle-income countries about what is known about the effects of those public health law for medicines financing and affordability, and how this evidence was measured.

### 4.1. Public Health Law Analysis

Public health infrastructure, such as establishing decision making bodies related to medicines affordability and financing are formalised in different ways. In this study we observed that some civil law countries (Ecuador, Ukraine) prescribe the structure, function, and responsibilities of these bodies in national law. Common law countries (Ghana, Philippines, South Africa) construct these types of public health infrastructure in policy rather than law [58]. For example, the national pharmaceutical policy in Ghana establishes the National Medicines Selection Committee and the National Medicines Price Committee; the latter committee sets prices for all reimbursed medicines [59]. Investigating optimal governance structures and models (centralisation vs. decentralisation) is an important component of the effective implementation of laws by the competent committees, authorities, and bodies. One study from Ecuador examined the impact of policy changes allowing hospital drug and therapeutic committees to select and procure non-essential cancer medicines on their utilisation in hospitals [35]. Another study from the Ukraine reported that there was no clear relationship between the Committee recently established to select essential medicines and the decision making process to expand the reimbursement list [31]. Further studies should be undertaken to evaluate the effect of changes in public health infrastructure and the relationships between different bodies related to the selection and use of medicines.

In our study, most outcome measures relate to economic policies and regulation such as the cost of medicines for patients. Future research should also focus on other types of outcome measures to reveal how different components of laws function in practice. Each country we studied has legal rules requiring public financing for a selection of pharmaceuticals, yet the effects of this legal intervention are not assessed in practice. Future research could quantify the level of government budget allocations (as was done in Ukraine) and/or actual spending on essential medicines provision/pharmaceutical benefits, or the real-time availability in health facilities of pharmaceuticals to which UHC members are entitled (also related to benefits selection).

Transparency and the provision of information is a key objective of public health law. In the context of medicines financing and affordability, transparency encompasses information disclosure by all actors to empower patients/users (to understand their medicines entitlements and compare prices), health professionals (to provide the best care at the most affordable price), and public authorities (to compare, set, and negotiate prices). In this study national law embeds more obligations for pharmaceutical sector stakeholders to provide information about medicines pricing than about pharmaceutical benefits. Future research should investigate the effectiveness of legal interventions at enhancing patient-level knowledge about their medicine’s entitlements and lower-priced alternatives. Further studies should also explore which conditions and factors facilitate health providers, public authorities, and economic operators to provide the information about medicines pricing and benefits that they are required to [58].

Health equity objectives, and specifically state obligations and individual rights, were identified in the laws of all countries in our study. Only one study examined the effect of a constitutional entitlement on the rate of medicines utilization in hospital [37]. Future research should consistently disaggregate outcome measures (i.e., patient prices, out-of-pocket payments) by wealth quintile or other measure to assess equitable financing. One example of this is the study from Ukraine that disaggregated many indicators (ex. number of pharmacies, proportion of population benefitting from medicines access, increase in consumption of medicines after legal intervention), by geographic region in the country [31].

Laws aiming to provide remedies and sanctions for rights violations are important for holding pharmaceutical sector stakeholders to account for their responsibilities and actions, particularly towards other objectives of public health law (i.e., providing information, governing through committees and bodies, promoting health equity, implementing economic incentives/disincentives and regulation). We identified one study that reported no informal complaints from pharmaceutical sector actors, and a second study that investigated formal court claims by patients for access to publicly funded medicines [29,31]. Future research should investigate two types of outcomes. Intermediate outcomes assess whether and how processes for seeking remedies and sanctions are used. These outcomes could include the number of patients/users who have filed a complaint for an alleged violation of their rights to available and affordable pharmaceutical benefits; number of healthcare providers or economic operators who have been reported for alleged violations such as failing to provide required price information or engaging in anti-competitive behaviour. End outcomes assess the final result of remedial action and/or sanctions, and these outcome measures can be paired with measures of other public health law objectives (i.e., trends in medicines prices or patients’ out-of-pocket payments) to build a more complete picture of how laws are influencing access to medicines in practice. Possible end outcomes could include the number and nature of sanctions actually applied to health providers and economic operators (i.e., fines for anticompetitive behaviour). Another end outcome could be the number and nature of administrative decisions made on patients’ complaints. Administrative and quasi-judicial remedies available to patients at the level of health facilities could help redress rights violations and avoid or ease the pressure on the judicial system in countries where patients litigate for medicines.

### 4.2. Role of Pharmaceutical Policy

Laws about pharmaceuticals are neither adopted nor implemented in a vacuum. They are influenced by and have an effect on domestic pharmaceutical policies and practices. It is therefore surprising that few papers we screened describe the legal environment (with regards to medicines access) in which the study takes place. A survey of 55 and 56 low- and middle-income countries found that national pharmaceutical policies are associated with lower antibiotic use and better use of medicines (based on a quality use of medicines composite index), respectively [60,61].

Pharmaceutical policies, although not legally binding, are governance instruments. They may direct and instruct certain priorities and actions in the national pharmaceutical sector, including the adoption or reform of legislation [38,62]. In this way policies can have direct and indirect effects on systems-level and/or patient-level access to medicines [40,60,62]. Pharmaceutical policy in some countries creates a supportive environment for the introduction of legislation for economic incentives/disincentives and regulation. For example, Ghana’s 3rd National Medicines Policy (2017) recommends a mix of interventions to achieve lower medicines prices. In 2017, legislation was adopted to exempt selected pharmaceutical raw materials and active pharmaceutical ingredients as well as selected imported finished pharmaceutical products from Value Added Tax (VAT). At the time of adoption, private sector stakeholders projected that the VAT exemptions on selected medicines would result in a 30% reduction in the NHIS tariffs on medicines [59,60,62].

### 4.3. Policy Implications

We argue that a greater understanding is needed of the tangible effects on access to medicines of adopting, implementing, and enforcing specific legal concepts and texts on the path towards UHC. Most existing guidance publications about pharmaceuticals for law makers regard pharmaceutical regulation (i.e., safety, quality, and effectiveness) and clinical trials, and high-level pharmaceutical sector policies (with little focus on UHC) [63,64,65]. Recent WHO guidance documents on pharmaceutical pricing strategies identify the need for legislation as ‘infrastructure’ to implement policies (ex. for pooled procurement, price transparency) [38,39,66]. However, these documents lack an analysis of the types of legal instruments, the legal concepts, examples and approaches to legislate for these interventions [38,39].

Overall, our analytical approach using a public health law framework can inform other governments in analysing legal reforms to identify strengths and gaps with respect to medicines financing and affordability.

To this end, our study includes one proposed legal act for National Health Insurance in South Africa. Our analysis reveals key strengths of this proposed legislation, being the establishment of committees responsible for determining pricing and medicines benefits, a range of economic policies and regulation for public financing, pooled financing, the selection of benefits, and equitable financing; the provision of information about pharmaceutical benefits by public authorities to patients; and the availability of remedies for users/patients and sanctions for public authorities. Our analysis shows that, in addition to the above measures, the proposed Act could embed a duty and mechanisms for public authorities to monitor medicines affordability and financing, economic incentives for medicines procurement in the context of national health insurance, and the dissemination of pricing information by pharmaceutical sector stakeholders. Similar analyses could be made for other MICs introducing legislation for UHC, such as the draft National Health Protection Act in Bangladesh.

### 4.4. Strengths & Limitations

The key strengths of this study are the diversity of middle-income countries and national contexts (from early to advanced UHC systems), which provide insights from different types of legal systems. Our study uses law as a primary source (rather than a key informant’s interpretation of the law/policy, as is done in other studies). In addition, this study interprets the findings with the benefit of insights of co-authors who are local pharmaceutical policy experts. Applying a public health law framework takes a broad view of all possible legal strategies for medicines financing and affordability (from infrastructure to sanctions), not only the substantive pricing and financing policies that most other studies focus on. Finally, this research highlights the empirical effects of known legal interventions, which is of interest to pharmaceutical policy and regulation researchers designing and conducting such studies, and legal scholars seeking to understand how the impact of laws can be measured.

Our study has several limitations. The overall aim of our study was not to produce a detailed description of the entire pharmaceutical policy landscape of each country. Therefore, aspects of medicines financing and affordability that are addressed in national pharmaceutical policies are only briefly described in this article.

Analyses of federal legislation excludes potentially important legal norms, developments, and contextual nuances at the regional/state/provincial, or municipal levels, which could play a role in countries such as the Philippines. From this study it is not possible to determine whether and how each federal law is implemented in practice. We enhance the reliability of our legal mapping by using explicit search terms, an automated and manual search, and classification of legal text and two coders who used a framework with categories and definitions.

This study includes relatively few evaluations of UHC and related legal reforms for medicines financing and affordability considering the sweeping UHC reforms that most countries in our sample have embarked on. In general, our screening process revealed a reasonable number of studies investigating the effect of a legal intervention on access to healthcare in general, but few specifically disaggregated healthcare into its subcomponents (i.e., medicines, services, etc.), thereby precluding us from generalising the effects on access to medicines specifically.

### 4.5. Future Research

Future studies of public health law interventions should prioritise robust study designs such as interrupted time series analysis and natural policy experiments from which conclusions about a causal relationship between a legal instrument and an indicator of access to medicines can be drawn. Theory-based qualitative studies can also be useful to understand the context, facilitators, and barriers to legal implementation and enforcement. Pharmaceutical law and policy research should draw from a range of disciplines including political science, economics, clinical medicine, public health, law, sociology, and anthropology.

Second, future studies should diversify the outcome measures used (including by investigating intermediate and end outcomes) and match them to the public health objectives of legislation. In addition to the above examples, we also propose investigating clinical and social outcomes (i.e., ability to pay one’s bills) in patients who receive free-of-charge access to pharmaceutical benefits (related to health equity) [67].

Third, future research should investigate the process and pace of UHC reform with regards to pharmaceuticals, specifically to understand how trust and buy-in should be built among pharmaceutical sector stakeholders in order to design and implement proposed UHC legislation. Legislation that does not enjoy broad stakeholder support will face many challenges in implementation.

Fourth, future research could explore the effect of international law and trade and investment treaties on domestic UHC and measures of access to medicines. Changes in international law or a State’s ratification of international law (and therefore having an obligation to bring national law, policy and practice in line with international norms) could have an effect on essential medicines [68].

Fifth, pharmaceutical sector stakeholders such as pharmaceutical and health insurance companies, and health providers, may introduce sector-specific rules and policies that affect measures of access to medicines. Industry initiatives can also have a significant impact on access to particular products or classes of medicines, such as for diabetes and Direct-Acting Antivirals for treating the hepatitis C virus [69,70,71,72]. Therefore, the interaction between UHC legislation and industry’s access policies is an important avenue for future research.

## 5. Conclusions

Five objectives of public health law were identified in national law for medicines financing and affordability in our five middle-income countries. Empirical evaluations of national law were mostly designed to evaluate economic policies and regulation, while scarcely evaluating the other four objectives of public health law (public health infrastructure, information, health equity, remedies and sanctions). Although laws for access to medicines are frequently adopted by law makers, the full range of their intentional and unintentional effects on medicines access in health systems is under studied. Adopting laws for all components of public health law and understanding their effectiveness at promoting universal access to medicines, is important to enforce UHC reforms. Further studies are required to understand the range of legal interventions for UHC and pharmaceuticals and to measure the spectrum of possible effects on access to medicines in middle-income countries. This analysis of legal interventions can provide future direction to design and analyse legal action and reforms initiated for improved access to medicines.

## Figures and Tables

**Table 1:** Overview of characteristics of the five middle-income countries.

	Ecuador	Ghana	Philippines	South Africa	Ukraine
World region	Americas	Africa	Western Pacific	Africa	Europe
Stage of UHC ^#^	Advanced	Advanced	Intermediate	Early	Advanced
Language of the national law database categorised by WHO [20]	Spanish	English	Filipino, English	English	Ukrainian, Russian, English
Population (millions), 2018 [22]	17.1	29.8	106.7	57.8	44.6
GNI per capita, Atlas method (current US$), 2018 [22]	6110	2130	3830	5750	2660
Legal system (categorised by WHO) [20]	Civil	Common Customary	Civil, Common	Civil, Common	Civil
Total fertility rate (births per woman), 2018 [22]	2.4	3.9	2.6	2.4	1.3
Infant mortality rate (per 1000 live births), 2019 [22]	12.0	33.9	21.6	27.5	7.2
Domestic public expenditure on health expenditure as a percent of general government expenditure (%), 2017 [23]	11.92	6.07	7.13	13.34	7.42
46.64	52.03	65.50	44.39	54.30
Out-of-pocket payments as a percent of total health expenditures (%), 2017 [23]	39.40	40.29	53.05	7.77	52.32
Total pharmaceutical sales as a percent of total health expenditure (%), 2014 [24]	16.30	25.00	24.40	11.90	36.20

^#^ Stages of UHC are defined as: Early = Legislative proposal for UHC covering vulnerable populations has not (yet) been adopted. Intermediate = Benefits package includes in-patient medicines only. Advanced = Benefits package includes in- and out-patient medicines.

**Table 2:** Five objectives of public health law (modified from Magnusson et al. [10]) in relation to medicines affordability and financing. Legend: * Economic operators are defined as business entities including but not limited to pharmaceutical manufacturers, distributors, importers, and wholesalers.

**Public health infrastructure**
These legal rules identify the public entitles (ex. Committees, agencies, authorities, bodies) responsible for actions related to the selection of essential medicines, or the supply/procurement, IP management, pricing, or reimbursement of medicines. This may include the selection criteria for members of these decision-making bodies, their structure and organisation, roles and responsibilities, and other governance aspects [10].
**Economic policies and regulation**
These legal rules aim to incentivise and regulate the behaviours of government agencies, businesses, professionals and individuals [10]. Economic policies and regulation may include regulations for financing health care and insurance through public budgets (including pooling financing); the criteria for selecting and reviewing pharmaceutical benefits under UHC, and the periodicity of revision; the procurement of pharmaceuticals by public agencies; and policies and formulas for granting patents, data exclusivity, or intellectual property flexibilities, and for identifying prohibited anti-competitive behaviour of economic actors.
**Transparency and the provision of information**
These legal rules aim to inform and educate third parties regarding a particular behaviour. In the context of medicines financing and affordability, transparency encompasses information disclosure by all actors to empower patients/users (to understand their medicines entitlements and compare prices), health professionals (to provide the best care at the most affordable price), and public authorities (to compare, set, and negotiate prices). Information for users or the public can include entitlements to a UHC benefits package, and how health services are financed and organised.
**Health equity**
These legal rules aim to recognise the rights of individuals and the duties on States to protect and promote those rights. Health equity may include the explicit recognition of: individuals’ rights to access essential medicines and/or essential medical products/goods; the role of States to respect, protect, and fulfil individual rights; Regulations for the financial coverage of poor people or people in vulnerable situations to protect individuals and households from (catastrophic) out-of-pocket payments.
**Remedies and sanctions**
These legal rules aim to create mechanisms to hold pharmaceutical sector stakeholders to account for their responsibilities and actions, particularly towards other objectives of public health law (i.e., providing information, governing through committees and bodies, promoting health equity, implementing economic incentives/disincentives and regulation). Remedies and sanctions may include: The availability of recourse for complaints and remedies for users in case of an alleged rights violation in relation to UHC and pharmaceuticals; Sanctioning the behaviour of health providers who are involved in the prescription, dispensing, and/or use of medicines, that is contrary to good pharmaceutical care and/or medical ethics; Sanctioning the behaviour of public authorities (including government officials and employees) that is inconsistent with their legal and ethical obligations regarding pharmaceutical care; Sanctioning the behaviour of economic operators * that is defined in legislation as undesirable.

**Table 3:** Overview of the national laws in five middle-income countries that embed the objectives of public health law for the affordability and financing of medicines. The laws on which this analysis is based are listed in Supplementary Material S3. Legend: X = objective in law. P = objective proposed in draft law. * = includes government officials and public employees.

Objectives of Public Health Law	Ecuador	Ghana	Philippines	South Africa	Ukraine
Infrastructure & governance (Decision making bodies for:)					
Essential medicines selection	X				X
Medicines pricing	X		X	X	X
Benefits selection	X	X		P	
Procurement	X	X	X	P	
IP management & competition	X		X	X	X
Economic policies & regulation					
Public financing	X	X	X	X and P	X
Pooled financing	X	X	X	P	X
Medicines pricing	X	X	X	X	X
Benefits selection	X	X	X	P	X
Procurement	X	X	X		X
IP management & competition	X	X	X	X	X
Information dissemination (about Pricing/about UHC Benefits)					
For users/public	X/	/X	X/	/P	
By health providers	X/		X/	X/	X/X
By public authorities	X/	/X	X/	X/P	X/X
By economic operators	X/			X/	
Health equity					
Individual rights	X	X	X	X	X
State obligations	X	X	X	X	X
Equitable financing	X	X	X	X and P	X
Remedies & sanctions					
Remedies for users/public	X	X	X	X and P	X
For health providers	X		X	X	X
For public authorities *	X	X	X	X	X
For economic operators	X		X	X	X

**Table 4:** Results of the scoping review. Evaluations of legal interventions for medicines financing and affordability in five middle income countries.

Article	Country	Study Design	Legal Intervention	Main Objectives of the Legal Intervention Studied (Definitions in Table 2)	Study Population & Data Source	Main Findings
1. Mena MB, 2020 [41]	Ecuador	Cross-sectional design describing characteristics of patient claiming government-funded access to cancer medicines in Ecuadorian courts. This study investigates the frequency of successful claims, the clinical eligibility of patients to receive the medicines they claim, the therapeutic monitoring of court-ordered medicines, and the potential budget impact of court-ordered medicines if extended to all eligible potential patients in Ecuador.	Constitution of the Republic of Ecuador. Asamblea Nacional de la Republica del Ecuador; 2008.	Health equity Sanctions & remedies	25 court claims (representing 33 patients with cancer) between 2012–2018 for government-funded cancer medicines. Data was collected from court decisions published on the official website of the Judicial System. Data from trial registration pages (National Library of Medicine, clintrials.gov) were used to assess whether the claimants fulfilled the clinical trials eligibility criteria. Therapeutic monitoring reports were sourced from the National Directorate of Medicines and Medical Devices. To estimate the potential budgetary impact administrative statistics from Ecuador were used. When they were not available, extrapolations to the Ecuadorian population were based on data from www.Orpha.net	97% of court claims (*n* = 32/33 court claims) involving the Ministry of Public Health for government-funded cancer medicines were granted between 2012–2018. 51.5% of patients did not meet the eligibility criteria used in key clinical trials demonstrating the efficacy of the requested medicine. Potential budgetary impact if medicine universalised to all patients who need; would consume 114.5% of Ecuador’s 2017 annual public pharmaceutical budget.
2. Eckhardt et al. 2019 [42]	Ecuador	Cross-sectional qualitative study to explore the perceived effects of the 2008 health reform implementation on rural primary health care services and financial access of the rural poor.	Constitution of the Republic of Ecuador. Asamblea Nacional de la Republica del Ecuador; 2008	Economic policies and regulation Health equity	Data was collected through focus group discussions with health staff, local health committee members, village leaders, and community health workers in a rural region of the province of Esmeraldas. Qualitative content analysis was applied and Walt and Gilson’s model for health policy analysis was used to interpret the results.	Obstacles in communication about the reform: 40–50% of villagers were aware that medicines are now free of charge; preconception that medicines in public sector are of unreliable quality; Increased demand for health care services: Reports of patients requesting medicines without being ill; Availability after the reform: more free medicines in the public system, but high patient loads sometimes caused competition for supply and shortages; Financial effects for the population: unconfirmed reports of free medicines being sold (not allowed).
3. WHO, 2019 [43]	Ukraine	Cross-sectional qualitative and quantitative design combined with a time series analysis of INN trends to assess whether the AMP had succeeded in fulfilling its objective to provide more patients with affordable medicines for selected chronic diseases (cardiovascular diseases, type 2 diabetes and bronchial asthma); and to evaluate possible uneven uptake of the programme across oblasts (geographic areas) and possible explanatory factors	Law on State Financial Guarantees of Health Care Services to the Population (2017)	Economic policies and regulation Health equity Infrastructure	Study population was the national pharmaceutical market, and local and national pharmaceutical stakeholders. Data sources were literature and legal documents, quantitative data from national and regional authorities and private companies about the pharmaceutical sector, and structured qualitative interviews with stakeholders. Data was collected from April-September 2018 for the period prior to and following the implementation of the 2017 law.	**Accessibility:** (a) >8 million Ukranians enrolled in/covered by the AMP; (b) 6–28 pharmacies per 100,000 inhabitants participated in the AMP, amounting to 40% of pharmacies in Ukraine; (c) variable but increased consumption of reimbursed medicines in all oblasts, ranging from from 8% to 50% in certain oblasts. **Affordability:** (a) 85% average reductions in patients’ co-payments; (b) prices decreased in all except one therapeutic area (bronchial asthma) covered by AMP; (c) budget allocation to AMP by the Ministry of Health was 21.8 million Eur in 2017 and 31 million Eur in 2018. **Acceptability:** No major complaints reported by pharmacies (regarding timely reimbursement by authorities) and patients (except that they must sometimes wait one day for their medicines). **Efficiency:** (a) INNs included in the AMP were 21 by April 2017 and 23 by end 2017; (b) Sales of medicines and their market share increased markedly after implementation. (c) No clear link between the AMP and the recently established Essential Medicines committee, nor is there a formal process for expanding the therapeutic areas covered by AMP.
4. Moodley et al., 2019 [44]	South Africa	Interrupted time series analysis, examined the impact of the in 2004 implemented Single Exit Price (SEP) intervention on private sector price data from a basket of generic medicines.	Act No. 101 of 1965 on Medicines and Related Substances Act, 1965 amended through 2002 Regulations for a transparent pricing system, 2004	Economic policies and regulation Information	Price data were obtained from pharmacy dispensing files, claims data and other routinely collected data for a basket of medicines (50 originator and their available generics). Data was obtained for the period December 1999-December 2014, corresponding to five years prior to the implementation of the SEP (1999–2003) and over the subsequent ten years (2004–2014).	Three trends were observed with different generics: (1) Medicine prices prior to 2004 showed a year-on-year steady rate of increase. The average rate of increase before the regulation was higher than the average rate of increase after the regulation. (2) Medicine prices were already decreasing prior to the intervention in 2004, and after intervention the medicine saw a price reduction (*n* = 28 generic medicines); (3) prices had a steady increase in price between 1999 to 2004 with a steep drop in 2004, which may be related to competition or stock issues (*n* = 3 generics).
5. Moodley et al., 2019 [45]	South Africa	Interrupted time series analysis evaluating the impact of the in 2004 implemented Single Exit Price (SEP) on the retail price of a basket of originator medicines.	Act No. 101 of 1965 on Medicines and Related Substances Act, 1965 amended through 2002 Regulations for a transparent pricing system, 2004	Economic policies and regulation Information	Private sector retail price of fifty originator medicines, based on WHO/HAI basket of medicines. Price data was obtained from pharmacy dispensing files, claims data, and other routinely collected data. Data was obtained for the period December 1999–December 2014.	“Most medicines investigated showed a smaller yearly increase in price compared to before regulations due to the controlled pricing environment introduced by Government.” Depending on which list the product appeared (global core, regional core, supplementary), the average price changed by 19.87% (SD 10.62%)–23.38% (SD 12.43%).
6. Kanmiki et al., 2019 [46]	Ghana	Longitudinal study examining changes in out-of-pocket payments vis-à-vis health insurance claims to investigate the impact of Ghana’s National Health Insurance Scheme (NHIS) on out-of-pocket healthcare payments (medicines, services, and obstetric care).	National Health Insurance Act, 2003. Act 650. Accra, Ghana. 2003 (revised to Act 852 in 2012)	Economic policy & regulations Health equity	Revenue data for out-of-pocket payments and health insurance claims for unspecified medicines, services and obstetric care. Data was collected for the years 2010–2014 from public primary healthcare facilities in seven districts of the Upper East Region of northern Ghana.	Between 2010–2014, out-of-pocket payment for medications in primary healthcare reduced by 62% and health insurance claims for medicines increased by 34% (2013) and 9% (2014).
7. Durán et al. 2019 [47]	Ecuador	Interrupted time series analysis investigated the impact of two policies on the utilization of new targeted oncologic medicines in Ecuador.	National policy (April 2012) “allowing hospital drug and therapeutic committees (DTCs) to select and procure drugs not previously included in the NEML” Policy (April 2013), the core of the policy being “the requirement that any decision taken by hospital DTCs regarding selection of new drugs must be confirmed by the National Medicines Directorate, an administrative unit of the Ministry of Health”.	Economic policy & regulations Infrastructure	Patient dispensing data for twenty-three targeted oncologic medicines over five years (2010–2014). Data is draw from routinely collected data from the six largest Ecuadorian cancer hospitals (three private and three public).	“Transferring the responsibility to select new drugs to hospital drug and therapeutic committees produced an increase in prescription intensity of targeted oncologic drugs, mainly in the private sector.” The second policy intervention aimed to centralise these decisions in a single body (while still keeping the first level of decision making in hospitals) reduced the incidence of prescriptions of targeted expensive oncologic medicines.
8. Dalinjong et al., 2018 [48]	Ghana	Convergent parallel mixed methods design, using qualitative and quantitative data investigating whether the free maternal health policy under the National Health Insurance Scheme (NHIS), [36] introduced in 2008, eliminated out-of-pockets payments for maternal health services, including medicines.	National Health Insurance Act, 2003. Act 650. Accra, Ghana. 2003 (revised to Act 852 in 2012). Implementing policy: Free maternal health policy (2008) under the NHIS.	Economic policy & regulations Health equity	406 women in the Kassena-Nankana municipality (rural area) who used pregnancy services, 25 midwives/nurses and 3 managers/directors. Data was collected through structured questionnaires (with women informants), 10 focus group discussions (with women informants), and in-depth interviews (with health providers and managers/directors). Data were collected in March-August 2016.	Half of women interviewed reported making direct out-of-pocket payments for medicines during pregnancy care. The average paid for medicines was US$18.10 (SD US$34.40).
9. Durán et al., 2018 [49]	Ecuador	Longitudinal study of hospital dispensing data examining utilization and expenditure trends of oncologic medicines (targeted, chemotherapy, hormonal) in 2010–2014.	Constitution of the Republic of Ecuador. Asamblea Nacional de la Republica del Ecuador; 2008.	Health equity	23 targeted oncologics, 43 chemotherapeutics, and 11 hormonal medicines prescribed to 40,099 between 2010 and 2014. 60.3% of patients were female. Data from three public and three private cancer centres in Ecuador, comprising the six largest Ecuadorian cancer hospitals in 2010–2014.	The proportion of patients using targeted medicines doubled in the period of 2010–2014, whilst the utilization of chemotherapy showed a downward trend, and the use of hormonal therapy remained stable with a dip in 2012. Total expenditures on cancer drugs more than doubled (by factor 2.3) in 5 years, although the total number of patients was rather stable through the period of analysis. The rising pattern is driven by the expenditures on targeted drugs.
10. Dalinjong et al., 2017 [50]	Ghana	Quantitative and qualitative convergent parallel mixed methods study, using structured questionnaires and focus group discussions estimating out-of-pocket payments and financial impact during childbirth under the free maternal care policy under the National Health Insurance Scheme (NHIS).	National Health Insurance Act, 2003. Act 650. Accra, Ghana. 2003 (revised to Act 852 in 2012) Implementing policy: Free maternal care policy (2008) under the NHIS.	Economic policy & regulations Health equity	353 women (mean age 27 years) who gave birth in health facilities “in one rural and poor area of Northern Ghana; the Kassena-Nankana municipality.” Data were collected in March-August 2016.	91.8% of women incurred a mean out-of-pocket payment for medicines during childbirth of US$24.70.
11. Ashigbie et al., 2016 [51]	Ghana	Qualitative cross-sectional study of key informants, investigating the challenges of medicines management in the public and private sector under the National Health Insurance Scheme (NHIS).	National Health Insurance Act, 2003. Act 650. Accra, Ghana. 2003 (revised to Act 852 in 2012)	Economic policy & regulations Health equity	Semi-structured interviews with 26 key informants purposively selected from public and private sector hospitals and standalone pharmacies (including mission hospitals), pharmaceutical supplies, and NHIS district offices in the Eastern, Greater Accra and Volta regions of Ghana, interviewed between July and August 2014.	Most informants “agreed that the introduction of the NHIS has increased access to and utilization of medicines by removing cost barriers for patients.” Common concerns include “delays in receiving NHIS reimbursements, and low reimbursement rates for medicines which result in providers asking patients to pay supplementary fees.” Differences between private and public sectors are weak separation of prescribing and dispensing and limited use of drugs and therapeutic committees in the private sector, the disproportionate effects of unfavourable reimbursement prices for medicines, and inadequate participation of the private sector providers (especially pharmacies and licensed chemical sellers) in the NHIS.”
12. Aryeetey et al., 2016 [52]	Ghana	Retrospective cross-sectional study investigating the effect of the National Health Insurance Scheme (NHIS) on the availability of essential medicines in mission health facilities.	National Health Insurance Act, 2003. Act 650. Accra, Ghana. 2003 (revised to Act 852 in 2012)	Economic policy & regulations	Structured questionnaires and exit interviews gauging the availability of selected essential medicines in 34 mission facilities (hospitals, clinics, specialist centres, and (primary) health centres), grouped into the three ecological zones: 12 coastal (southern), 17 forest (middle) and 5 savannah (northern). Data were collected for the periods 2003 and 2010.	Availability of essential medicines in facilities generally improved after the introduction of the NHIS in all three ecological zones.
13. Espinosa MV, 2016 [53]	Ecuador	Pre-/post-intervention study of medicines ceiling prices of cardiovascular medicines after the implementation of a price control regulation.	Executive Decree no. 400. Regulation of medication price setting. 2014, modified 2017	Economic policy & regulations	364 cardiovascular medicines marketed in Ecuador’s private sector were included. Data was collected from administrative datasets. Pre-intervention sales prices were derived from the Ecuadorian Pharmacotherapeutic Formulary. Post-intervention sales prices were the ceiling prices set by the Ecuadorian Technical Secretariat for fixing prices.	There was no significant average change in the ceiling price of the 364 cardiovascular medicines with prices regulated under the Executive Decree no 400. However, the price of thiazides and loop diuretics increased by US$0.018 per unit (tablet) (*p* = 0.02), statins decreased by US$0.21/unit (*p* = 0.001), and angiotensin-converting enzyme inhibitors decreased by US$0.10/unit (*p* = 0.014) after the regulation took effect.
14. Kusi et al., 2015 [54]	Ghana	Cross-sectional representative household survey investigating the effect of the National Health Insurance Scheme (NHIS) on out-of-pocket health expenditures (OOPHE), comparing expenditures from insured and uninsured persons.	National Health Insurance Act, 2003. Act 650. Accra, Ghana. 2003 (revised to Act 852 in 2012).	Economic policy & regulations Health equity	Out of pocket health expenditures of 1082 household members who reported sick in the last four weeks. (449 were uninsured, 633 were insured) Data was collected through a representative household survey from three districts in Ghana between February-April 2011.	The NHIS significantly decreased OOPHE, but with respect to the cost of prescribed drugs bought from outside the facility, paradoxically insured persons paid a higher amount (US$9.51) than uninsured persons (US$7.08), an observation for which the authors did not have an explanation.
15. De Guzman et al., 2014 [55]	Philippines	Qualitative cross-sectional study investigating the knowledge, attitudes, and practices with respect to the Cheaper Medicines Act (CMA) and the Government Mediated Access Price (GMAP) list, assessing the impact and implementation of the CMA among households	Republic Act 9502, “Universally accessible and quality medicines act of 2008 (“Cheaper Medicines Act of 2008”). Congress of thePhilippines,” 2008. Executive Order No 821 S, “Prescribing the maximum drug retail prices for selected drugs and medicines that address diseases that account for the leading causes of morbidity and mortality. Philippine Government, 2009.	Economic policies & regulation Information	62 female respondents residing in metro Manila from three socio-economic classes (SECs) participated in 9 focus group discussions conducted in June 2013. Participants were selected based on the Philippine Marketing and Opinion Research Society (MORES) classification and on their being household (HH) decision makers on health matters	“Across all SECs, there is low spontaneous awareness of the CMA although many [respondents are] spontaneously aware of the Generics Act.” “Across all SECs, mass media channels are main sources of awareness and information on the CMA. Government doctors and health centres are poor sources of information on the CMA but are very good sources of information and advice on generics especially among the lowest SEC. Private doctors are poor sources of information on the CMA and generics.” “Respondents across all SECs have not noticed the GMAP price list in drugstores. They also have not noticed price reductions in branded drugs-possibly because GMAP does not cover drugs they buy including those for common ailments.”
16. Sarol, Jr., 2014 [56]	Philippines	Cohort design study investigating the impacts of the maximum drug retail pricing (MDRP) policy and the government-mediated access prices (GMAP) policy, flowing from the “Cheaper Medicines Act” of 2008, on selected medicines molecules directly affected by the MDRP/GMAP polices.	Republic Act 9502, “Universally accessible and quality medicines act of 2008 (“Cheaper Medicines Act of 2008”). Congress of the Philippines,” 2008. Executive Order No 821 S, “Prescribing the maximum drug retail prices for selected drugs and medicines that address diseases that account for the leading causes of morbidity and mortality. Philippine Government, 2009.	Economic policies and regulation	Price data of eleven selected medicine molecules which were placed under MDRP/GMAP listing. Data was collected through independent surveys conducted by IMS Health Philippines from a stratified sample of 600 retail medicine stores in 2009 and 2011 each. Price data were obtained using a mystery shopper approach.	Ten of the 11 medicines significantly decreased in mean price by being listed as MDRP/GMAP reference drugs. However, the author concluded that the number of MDRP/GMAP listed medicines was very limited compared to the total list of essential medicines and the polices may not have had a tangible effect yet.
17. Nguyen et al., 2011 [57]	Ghana	Cross-sectional study evaluating the impact of the National Health Insurance Scheme (NHIS) on households’ out-of-pocket spending and catastrophic health expenditure two years after the introduction of NHIS	National Health Insurance Act, 2003. Act 650. Accra, Ghana. 2003 (revised to Act 852 in 2012)	Economic policy & regulations Health equity	Health expenditure data from a survey of 2500 households (11,617 persons in total, of which 6718 are NHIS non-members and 4899 are NHIS members). The survey was conducted in two rural districts, Nkoranza and Offinso, in September–October 2007.	NHIS members incurred out-of-pocket medicine payments that equaled 73% of those incurred by NHIS non-members. A ‘not trivial’ portion of these out-of-pocket payments were for medicines (among other services) that should be covered by insurance.

## Supplementary Materials

The following are available online at https://www.mdpi.com/1660-4601/17/24/9524/s1, Supplementary Material S1: Search Strategies. Supplementary Material S2: Search results from academic databases. Supplementary Material S3: National legislation included in the study. Supplementary Material S4: Summaries of national legislation for five middle-income countries.
