# Supplementary material for: Essential Medicines in Universal Health Coverage: A Scoping Review of Public Health Law Interventions and How They Are Measured in Five Middle-Income Countries
Source: Int J Environ Res Public Health. 2020 Dec 18;17(24):9524. doi: 10.3390/ijerph17249524 (PMC7765934; doi:10.3390/ijerph17249524)
Supplement: Supplementary file 1 [file ijerph-17-09524-s001.pdf]

International Journal of
Environmental Research and Public Health

MDPI

Supplementary Materials

Supplementary Material S1. Search Strategies

List of national websites

Ecuador: National Assembly of the Republic of Ecuador (http://archivobiblioteca.asambleanacional.gob.ec); National Service of Public Procurement (https://portal.compraspublicas.gob.ec/sercop/biblioteca/); National Council of Health (http://www.conasa.gob.ec/); Ministry of Public Health of Ecuador (www.salud.gob.ec).

Ghana: Parliament of Ghana (http://ir.parliament.gh/handle/123456789/1409); Republic of Ghana Judiciary (http://www.judicial.gov.gh/index.php/fundamental-human-rights-and-freedom); Ghana Business Regulatory Reforms Portal (https://www.bcp.gov.gh/new/reg_details.php?id=MzI=)

Indonesia: National Agency of Drug and Food Control (https://jdih.pom.go.id); Ministry of Health (http://p2ptm.kemkes.go.id); Directorate General of Pharmaceutical and Health Equipment legal documentation and information network, Ministry of Health (https://farmalkes.kemkes.go.id/peraturan/); BPJS Health (https://bpjs-kesehatan.go.id/bpjs/); (Bureau of Law and Organization, Ministry of Health (http://hukor.kemkes.go.id).

Jordan: Ministry of Health laws and regulations repository (https://www.moh.gov.jo/Pages/viewpage.aspx?pageID=164).

Philippines: Official Gazette (https://www.officialgazette.gov.ph).

South Africa: South African Government documents search engine (https://www.gov.za/document).

Ukraine: Parliament of the Ukraine (https://zakon.rada.gov.ua/laws/main/index)

Ovid MEDLINE: Epub Ahead of Print, In-Process & Other Non-Indexed Citations, Ovid MEDLINE® Daily and Ovid MEDLINE® <1946-Present>

Search history sorted by search number ascending

#	Searches	Results	Type
1	exp Drugs, Essential/	842	Advanced
2	Prescription Drugs/	5831	Advanced
3	Insurance, Pharmaceutical Services/	3977	Advanced
4	(pharmaceutical* or drug* or medicine* or medication*).ti,ab,tw.	2291555	Advanced
5	((pharmaceutical* or medicine* or drug* or medication*) adj3 (policy or policies or law* or legislation or regulation)).tw,ti,ab.	10139	Advanced
6	Legislation, Drug/	10162	Advanced
7	"Drug and Narcotic Control"/	8725	Advanced
8	universal health coverage.ti,ab,tw.	2220	Advanced
9	universal health?care.ti,ab,tw.	435	Advanced
10	universal health system.ti,ab,tw.	58	Advanced
11	national health insurance*.ti,ab,tw.	10582	Advanced
12	universal coverage scheme*.ti,ab,tw.	61	Advanced
13	community-based health insurance*.ti,ab,tw.	214	Advanced
14	community based health insurance*.ti,ab,tw.	214	Advanced
15	medical services insurance*.ti,ab,tw.	17	Advanced
16	medical service insurance*.ti,ab,tw.	4	Advanced
17	medical assistance plan.ti,ab,tw.	4	Advanced

22	green card.ti,ab,tw.	25	Advanced
23	Jaminan Kesehatan.ti,ab,tw.	9	Advanced
24	Jamkesmas.ti,ab,tw.	7	Advanced
25	Jamkesda.ti,ab,tw.	0	Advanced
29	PhilHealth.ti,ab,tw.	15	Advanced
41	civil health insurance*.ti,ab,tw.	0	Advanced
42	exp Turkey/	34256	Advanced
43	exp Bangladesh/	10780	Advanced
44	exp Ukraine/	15878	Advanced
47	exp Indonesia/	10271	Advanced
48	exp Philippines/	8261	Advanced
49	exp Colombia/	10224	Advanced
51	exp South Africa/	41451	Advanced
53	exp Ecuador/	3681	Advanced
60	exp Colombia/	10224	Advanced
61	exp Ghana/	8031	Advanced
62	exp Jordan/	4149	Advanced
72	1 or 2 or 3 or 4 or 5 or 6 or 7	2301761	Advanced
78	8 or 9 or 10 or 11 or 12 or 13 or 14 or 15 or 16 or 17 or 22 or 23 or 24 or 25 or 29 or 41 or 39 or 40	13437	Advanced
79	42 or 43 or 44 or 47 or 48 or 60 or 51 or 53 or 61 or 62	145718	Advanced
80	72 and 78 and 79	85	Advanced

Embase Classic+Embase <1947 to 2020 May 15>.

Search history sorted by search number ascending

#	Searches	Results	Type
1	exp Drugs, Essential/	1276	Advanced
2	Prescription Drugs/	7981	Advanced
3	Insurance, Pharmaceutical Services/	127221	Advanced
4	(pharmaceutical* or drug* or medicine* or medication*).ti,ab,tw.	3452830	Advanced
5	((pharmaceutical* or medicine* or drug* or medication*) adj3 (policy or policies or law* or legislation or regulation)).tw,ti,ab.	15275	Advanced
6	Legislation, Drug/	14830	Advanced
7	"Drug and Narcotic Control"/	11850	Advanced
8	universal health coverage.ti,ab,tw.	2274	Advanced
9	universal health?care.ti,ab,tw.	601	Advanced
10	universal health system.ti,ab,tw.	72	Advanced
11	national health insurance*.ti,ab,tw.	15579	Advanced
12	universal coverage scheme*.ti,ab,tw.	79	Advanced
13	community-based health insurance*.ti,ab,tw.	225	Advanced
14	community based health insurance*.ti,ab,tw.	225	Advanced
15	medical services insurance*.ti,ab,tw.	16	Advanced
16	medical service insurance*.ti,ab,tw.	5	Advanced
17	medical assistance plan.ti,ab,tw.	5	Advanced

22	green card.ti,ab,tw.	46	Advanced
23	Jaminan Kesehatan.ti,ab,tw.	22	Advanced
24	Jamkesmas.ti,ab,tw.	9	Advanced
25	Jamkesda.ti,ab,tw.	0	Advanced
29	PhilHealth.ti,ab,tw.	38	Advanced
41	civil health insurance*.ti,ab,tw.	0	Advanced
42	exp Turkey/	0	Advanced
43	exp Bangladesh/	16263	Advanced
44	exp Ukraine/	16812	Advanced
47	exp Indonesia/	18425	Advanced
48	exp Philippines/	12055	Advanced
49	exp Colombia/	19744	Advanced
51	exp South Africa/	51980	Advanced
53	exp Ecuador/	5441	Advanced
60	exp Colombia/	19744	Advanced
61	exp Ghana/	11781	Advanced
62	exp Jordan/	6713	Advanced
72	1 or 2 or 3 or 4 or 5 or 6 or 7	3574489	Advanced
78	8 or 9 or 10 or 11 or 12 or 13 or 14 or 15 or 16 or 17 or 22 or 23 or 24 or 25 or 29 or 41 or 39 or 40	18752	Advanced
79	42 or 43 or 44 or 47 or 48 or 60 or 51 or 53 or 61 or 62	154766	Advanced
80	72 and 78 and 79	364	Advanced

Web of Science, searched on 18 may 2020

Set	Results		Edit Sets	Combine Sets
# 4	265	#3 AND #2 AND #1<br><br>Indexes=SCI-EXPANDED, SSCI, A&HCI, CPCI-S, CPCI-SSH, BKCI-S, BKCI-SSH, ESCI Timespan=All years	Edit	
# 3	44,744	TS=(universal health coverage) or TS=(universal health system) OR TS=(universal health?care) OR TS=(national health insurance*) OR TS=(universal coverage scheme*) OR TS=(community?based health insurance*) OR TS=(medical service* insurance*) OR TS=(medical assistance plan) OR TS=(green card) OR TS=(Jaminan Kesehatan) OR TS=(Jamkesmas) OR TS=(Jamkesda) OR TS=(PhilHealth) OR TS=(plan obligatorio) OR TS=(POS) OR TS=(civil health insurance*)<br><br>Indexes=SCI-EXPANDED, SSCI, A&HCI, CPCI-S, CPCI-SSH, BKCI-S, BKCI-SSH, ESCI Timespan=All years	Edit	
# 2	2,670,420	TS=(pharmaceutical* or drug* or medicine* or medication*)<br><br>Indexes=SCI-EXPANDED, SSCI, A&HCI, CPCI-S, CPCI-SSH, BKCI-S, BKCI-SSH, ESCI Timespan=All years	Edit	

# 1	524,241	TS=(philippine* or "south africa" or ecuador or indonesia or jordan or colombia or turkey or ukraine or bangladesh or ghana)<br><br>Indexes=SCI-EXPANDED, SSCI, A&HCI, CPCI-S, CPCI-SSH, BKCI-S, BKCI-SSH, ESCI Timespan=All years	Edit

Scopus – searched on 18 May 2020

	Search terms	Hits
#1	(TITLE-ABS-KEY (philippine* OR "south africa" OR ecuador OR indonesia OR jordan OR colombia OR turkey OR ukraine OR bangladesh OR ghana)) AND (TITLE-ABS-KEY (pharmaceutical* OR drug* OR medicine* OR medication*)) AND ((TITLE-ABS-KEY ((universal AND health AND coverage)) OR TITLE-ABS-KEY ((universal AND health AND system)) OR TITLE-ABS-KEY ((universal AND health?care)) OR TITLE-ABS-KEY ((national AND health AND insurance*)) OR TITLE-ABS-KEY ((universal AND coverage AND scheme*)) OR TITLE-ABS-KEY ((community?based AND health AND insurance*)) OR TITLE-ABS-KEY ((medical AND service* AND insurance*)) OR TITLE-ABS-KEY ((medical AND assistance AND plan)) OR TITLE-ABS-KEY ((green AND card)) OR TITLE-ABS-KEY ((jaminan AND kesehatan)) OR TITLE-ABS-KEY ((jamkesmas)) OR TITLE-ABS-KEY ((jamkesda)) OR TITLE-ABS-KEY ((philhealth)) OR TITLE-ABS-KEY ((plan AND obligatorio)) OR TITLE-ABS-KEY ((pos)) OR TITLE-ABS-KEY ((civil AND health AND insurance*)))))	613 document results
#2	(TITLE-ABS-KEY ((universal AND health AND coverage)) OR TITLE-ABS-KEY ((universal AND health AND system)) OR TITLE-ABS-KEY ((universal AND health?care)) OR TITLE-ABS-KEY ((national AND health AND insurance*)) OR TITLE-ABS-KEY ((universal AND coverage AND scheme*)) OR TITLE-ABS-KEY ((community?based AND health AND insurance*)) OR TITLE-ABS-KEY ((medical AND service* AND insurance*)) OR TITLE-ABS-KEY ((medical AND assistance AND plan)) OR TITLE-ABS-KEY ((green AND card)) OR TITLE-ABS-KEY ((jaminan AND kesehatan)) OR TITLE-ABS-KEY ((jamkesmas)) OR TITLE-ABS-KEY ((jamkesda)) OR TITLE-ABS-KEY ((philhealth)) OR TITLE-ABS-KEY ((plan AND obligatorio)) OR TITLE-ABS-KEY ((pos)) OR TITLE-ABS-KEY ((civil AND health AND insurance*)))	102,828 document results
#3	TITLE-ABS-KEY (pharmaceutical* OR drug* OR medicine* OR medication*)	9,951,576 document results
#4	TITLE-ABS-KEY (philippine* OR "south africa" OR ecuador OR indonesia OR jordan OR colombia OR turkey OR ukraine OR bangladesh OR ghana)	664,689 document results

Supplementary Material S2. Search results from academic databases

Results for 10 countries (7 study countries + Bangladesh, Colombia and Turkey)

Ovid Medline: 85 articles
Embase: 364 articles
Scopus: 613 articles
Web of Science: 265 articles
Total imported into Mendeley: 1327 articles
Total exported from Mendeley after automatic & manual deduplication: 905 articles
Total after duplicates automatically detected and manually resolved in Rayyan: 889 articles

Supplementary Material S3. National legislation included in the study

Ecuador

Ecuador's Constitution of 2008 with Amendments through 2015

LAW OF GENERIC MEDICINES FOR HUMAN USE 2005, modified 2011

ORGANIC CODE OF THE SOCIAL ECONOMY OF KNOWLEDGE, CREATIVITY AND INNOVATION , 2016

ORGANIC HEALTH LAW No. 67, modified 2015

ORGANIC LAW OF CONSUMER DEFENCE No. 21, 2000, reformed 2011

ORGANIC LAW OF THE NATIONAL HEALTH SYSTEM (Law No. 2002-80)

ORGANIC LAW OF THE NATIONAL SYSTEM OF PUBLIC PROCUREMENT 2008, modified 2018

ORGANIC LAW OF THE REGULATION AND CONTROL OF MARKET POWER, 2011

REGULATION OF MEDICATION PRICE SETTING FOR USE AND HUMAN CONSUMPTION . Executive Decree 400. 2014, modified 2017

REGULATION OF THE ORGANIC SYSTEM LAW NATIONAL PUBLIC PROCUREMENT . Executive Decree 1700, 2009, modified 2020

REGULATION OF THE ORGANIC LAW OF THE SYSTEM NATIONAL HEALTH . Executive Decree 3611, 2003

PRESIDENTIAL DECREE No. 1125 REFORMING THE ORGANIC LAW ON GENERIC MEDICINES FOR HUMAN USE, 2012

INSTRUCTIONS TO AUTHORIZE THE ACQUISITION OF DRUGS THAT DO ARE NOT IN THE TABLE OF BASIC MEDICINES FOR ESTABLISHMENTS IN THE INTEGRAL PUBLIC HEALTH NETWORK 2013

Ghana

Ghana's Constitution of 1992 with Amendments through 1996

National Health Insurance Act No. 852, 2012

Public Health Act No. 851, 2012

Patents Act, 2003 (ACT 657)

Public Procurement Act, 2003 (Act 663), amended in 2016 (914)

Appropriation Act, 2018

Value Added Tax (VAT) Exemption Legislative Instrument (LI) 2255 of Nov 2017

Indonesia

Indonesia's Constitution of 1945, Reinstated in 1959, with Amendments through 2002

LAW No. 24 YEAR 2011 CONCERNING THE SOCIAL SECURITY ADMINISTRATIVE BODY

LAW No. 36 OF 2009 ABOUT HEALTH

LAW No. 40 OF 2004 ABOUT NATIONAL SOCIAL GUARANTEE SYSTEM

LAW No. 13 of 2016 ABOUT PATENTS

DECREE OF THE MINISTER OF HEALTH No. 189 / MENKES / SK / III / 2006 about NATIONAL MEDICINE POLICY

DECREE OF THE MINISTER OF HEALTH No. HK.01.07 / MENKES / 176/2019 ABOUT

NATIONAL COMMITTEE FOR THE DEVELOPMENT OF THE NATIONAL FORMULARIUM

DECREE OF THE MINISTER OF HEALTH NO. 312 / MENKES / SK / IX / 2013 ABOUT 2013 NATIONAL ESSENTIAL DRUG LIST

DECREE OF THE MINISTER OF HEALTH No. HK.01.07 / MENKES / 395/2017 ABOUT LIST OF NATIONAL ESSENTIAL MEDICINE

REGULATION OF THE MINISTER OF HEALTH No. 6 of 2016 ABOUT ORIGINAL HERBAL MEDICINE FORMULARIUM OF INDONESIA

DECREE OF THE HEAD OF DRUG AND FOOD SUPERVISORY AGENCY No. HK.04.1.23.04.16.1769 2016

DECREE OF THE MINISTER OF HEALTH No. HK.01.07 / MENKES / 813/2019 ABOUT NATIONAL FORMULARIUM

REGULATION OF THE MINISTER OF HEALTH No. 54 OF 2018 ABOUT PREPARATION AND APPLICATION IN THE NATIONAL FORMULARIUM HEALTH GUARANTEE PROGRAM

REGULATION OF THE PRESIDENT No. 25 YEAR 2O2O ABOUT GOVERNANCE OF SOCIAL GUARANTEE AGENCY

REGULATION OF THE MINISTER OF HEALTH No. HK.02.02 / MENKES / 068 / I / 2010 ABOUT USING GENERIC MEDICINE IN GOVERNMENT HEALTH SERVICES FACILITIES

REGULATION OF THE MINISTER OF HEALTH No. 98 of 2015 ABOUT PROVISION OF THE HIGHEST DRUG PRICE INFORMATION

REGULATION OF THE PRESIDENT No. 12 OF 2013 ABOUT HEALTH INSURANCE

REGULATION OF THE PRESIDENT OF THE REPUBLIC OF INDONESIA NUMBER 111 OF 2013 ABOUT CHANGES TO PRESIDENTIAL REGULATION NUMBER 12 OF 2013 CONCERNING HEALTH GUARANTEES

REGULATION OF THE MINISTER OF HEALTH No. 28 of 2014 ABOUT OPERATING PROCEDURE NATIONAL HEALTH WARRANTY PROGRAM

REGULATION OF THE MINISTER OF HEALTH No. 51 OF 2018 ABOUT CHARGING OF FEES AND DIFFERENCES IN COSTS IN THE HEALTH GUARANTEE PROGRAM

REGULATION OF THE PRESIDENT No. 64 YEAR 2O2O ABOUT SECOND AMENDMENT TO PRESIDENTIAL REGULATION NUMBER 82 YEAR 2OI8 ABOUT HEALTH GUARANTEE

REGULATION OF THE PRESIDENT No. 82 YEAR 20 18 ABOUT HEALTH INSURANCE

REGULATION OF THE MINISTER OF HEALTH OF THE REPUBLIC OF INDONESIA NUMBER 52 OF 2016 ABOUT STANDARD RATES OF HEALTH SERVICES IN THE OPERATION OF HEALTH GUARANTEE PROGRAMS

REGULATION OF THE MINISTER OF HEALTH OF THE REPUBLIC OF INDONESIA NUMBER 69 OF 2013 ABOUT STANDARD RATES OF HEALTH SERVICES IN THE FIRST LEVEL OF HEALTH FACILITIES AND CONTINUED HEALTH FACILITIES IN THE OPERATION OF HEALTH GUARANTEE PROGRAMS

REGULATION OF THE MINISTRY OF LAW AND HUMAN RIGHTS NO. 39 OF 2018 CONCERNING PROCEDURES ON GRANTING OF COMPULSORY PATENT LICENSES

Jordan

Jordan's Constitution of 1952 with Amendments through 2016

Planning Law of 1971, LAW NO. 68 FOR THE YEAR 1971

Law for the unified procurement system for medicinal and drugs health medications and its modifications No. 91 of 2002

Public Health Law No. (47) of 2008

Regulation No. 28 of 2019 (Governmental Procurement System of 2019)

Civil Health Insurance System No. (83) for the year 2004 - Issued under Paragraph (C) of Article (66) of the Public Health Law No. 54 of 2002

Regulation No. (36) for the year 2008 / the system of administrative organization of the Ministry of Health

Competition Law No. (33) of 2004, amended by Amending Competition Law No. (18) of 2011

Patent Law No. 16, amended by Patent Law No. 17 of 2017

Generalization of the Prime Minister, Handling cases related to lawsuits

Regulation No. (83) for the year 2004 on civil health insurance system

Instructions for emergency cases No. (8) of 2004

Instructions for workers in any company or institution to be covered by civil health insurance No. (83) for the year 2004

Instructions for adding the physically disabled user No. (2) for the year 2004 In accordance with article 8, 38 of Regulation No. (83) for the year 2004 on civil health insurance system

Instructions for cases in which it is permissible to fulfil fees for treatment and prices for medicines from the participant, the uninitiated, the handicapped, or any benefactor with them, and the blood donor who holds the organ donor card No. (2) for the year 2004

Instructions for the participation of daily workers in the health insurance fund of No. (1) for the year 2004

Instructions for cases and diseases not covered by the health insurance fund No. (5) for the year 2004

Instructions for Maternity, childhood and family planning services No. (3) for the year 2004 and Maternity services, childhood and family planning, Instructions No. 3 of 2008

Instructions for the inclusion of individual citizens in civil health insurance No. (5) for the year 2004 in accordance with article 8B of Regulation No. (83) for the year 2004 on civil health insurance system

Instructions for the category of pregnant women to be covered by civil health insurance No. (9) of 2006

Instruction for determining of the income of Jordanian who is not holding not capable card in accordance with article 32 of Regulation No. (83) for the year 2004 on civil health insurance system

Instructions No. (7) of the year 2004 / Availability of drug in Hospitals and clinics issued with accordance with Article 23 D8B of Regulation No. (83) for the year 2004 on civil health insurance system

Instructions for treating the people of the Gaza Strip in hospitals and Ministry of Health centres No. (17) of 2015

Instructions No. (5) for the year 2004 for the Foundations and terms of processing and transfer of civilians covered by the category of social safety net in the poorest areas and the remote areas category

Principles of drug pricing for the year 2016 and its amendments in 2020 - Issued under Article (5) of the Law on Medicines and Pharmacy and its Amendments No. 12 of 2013 and Article (7) of the General Food and Drug Corporation Law No. 41 of 2008

Principles of dealing with requests submitted to the Hashemite Royal Court regarding exemption from medical treatment expenses

Philippines

Philippines's Constitution of 1987

Republic Act No. 10606 AMENDING REPUBLIC ACT NO. 7875, OTHERWISE KNOWN AS THE "NATIONAL HEALTH INSURANCE ACT OF 1995"

REPUBLIC ACT No. 10918, The Pharmacy Law

Republic Act No. 11223, The Universal Health Care Act of 2018

Republic Act No. 7394, THE CONSUMER ACT OF THE PHILIPPINES of 1992

Republic Act No. 7581, THE PRICE ACT of 1992

Republic Act No. 9184, THE GOVERNMENT PROCUREMENT REFORM ACT of 2002

Republic Act No 9502, THE Universally Accessible Cheaper and Quality Medicines Act of 2008

The Revised Implementing Rules and Regulations of Republic Act No. 9184 of 2016, otherwise known as the Government Procurement Reform Act

The Revised Implementing Rules and Regulations of the National Health Insurance Act of 2013 (RA 7875 as amended by RA 9241 and 10606)

South Africa

South Africa's Constitution of 1996 with Amendments through 2012

Act No. 12 of 2013: National Health Amendment Act, 2013

Act No. 18 of 2018: Competition Amendment Act, 2018

No. 68 of 2008: Consumer Protection Act, 2008

Act No. 101 of 1965: Medicines and Related Substances Act, 1965 amended through 2002

Proposed National Health Insurance Bill

Regulations relating to a transparent pricing system for medicines and scheduled substances, 2004

Ukraine

Ukraine's Constitution of 1996 with Amendments through 2019

Law No. 2168-VIII of 2017, 2020 About the state financial guarantees of medical service of the population

Law No. 123.96 of 1996, 2020 About medicines

Law No. 2801-XII of 1992, 2020 Fundamentals of Ukrainian legislation on health care

Law No. 3552-XII of 1993, 2020 About the status of war veterans, guarantees of their social protection

Law No.796-XII of 28 February 1992, 2019, On the Status and social protection of citizens affected by the Chernobyl disaster

Law No.875-XII of 21 March 1991 (with amendments. Edition of 13 February 2020), On the Basics of social protection of persons with disabilities in Ukraine

Law No.2402-III of 26 April 2001 (with amendments. Edition of 09 august 2019), On Protection of childhood

Law No.1584-III of 23 March 2000 (with amendments. Edition of 11 October 2018), On Victims of Nazi persecution

Law No. 3721-XII of 16 December 1993 (with amendments. Edition of 01 January 2020). On the basic principles of social protection of labor veterans and other elderly citizens in Ukraine

Law No. 1223-VII of 17 April 2014 (with amendments. Edition of 04 November 2018), On the restoration of the rights of persons deported on national grounds

Law No. 962-XII of 17 April 1991 (with amendments. Edition of 02 January 2020), on the rehabilitation of victims of repressions of the communist totalitarian regime of 1917-1991

Law No. 239/95-BP of 23 June 1995 (with amendments. Edition of 28 June 2015), On the donation of blood and blood components

Law No. 203/98-BP of 24 March 1998 (with amendments. Edition of 31 August 2018), On the Status of veterans of military service, law enforcement agencies and some other persons and their social protection

Law No. 9 of 2016 About public procurement

CABINET OF MINISTERS OF UKRAINE DECREE of August 17, 1998 № 1303 About streamlining of free and preferential release of medicines

CABINET OF MINISTERS OF UKRAINE DECREE from February 27, 2019 № 135 Some issues of drug reimbursement

CABINET OF MINISTERS OF UKRAINE DECREE from March 28, 2018 № 271 Some issues of the Public Oversight Board at the National Health Service

CABINET OF MINISTERS OF UKRAINE DECREE of March 25, 2009 № 333 Some issues of state regulation of prices for medicines and medical devices

CABINET OF MINISTERS OF UKRAINE DECREE of March 5, 2014 № 73 The implementation of a pilot project on the introduction of state regulation of prices for insulin drugs

CABINET OF MINISTERS OF UKRAINE DECREE of November 3, 2010 № 996 On ensuring public participation in the formation and implementation of public policy

MINISTRY OF HEALTH OF UKRAINE ORDER 11.02.2016 № 84 On approval of the Regulations on the National List of Essential Medicines and the Regulations on the Expert Committee on the Selection and Use of Essential Medicines

POSITION on the expert committee on the selection and use of essential medicines, 2016, 2017

Supplementary Material S4. Summaries of National Legislation for Five Middle-Income Countries

Supplementary Material E includes brief country summaries that illustrate the legal and policy landscape governing the affordability and financing of essential medicines.

Ecuador

The National List of Basic Medicines (National Essential Medicines List) is prepared by the Medicines & Supplies Commission (within the National Health Council) in accordance with WHO nomenclature. (ECU Regulation of the Organic Law of NHS art 41; ECU Organic Law of NHS) Public sector entities responsible for health benefits must exclusively purchase generic medicines (when available) in the National List of Basic Medicines. Exceptions to this rule include when the brand-name medicines of comparable quality is offered at a lower price than the generic product, or in the event of a health emergency. (ECU Law of Generic Medicines, art. 6) Health professionals are obliged to prescribe by both brand and generic name and those who fail to do so can be sanctioned. (Law of Generic Medicines, art. 14, 23)

The National Council for Fixing and Reviewing Prices for Medicines for Human Use, attached to the Ministry of Public Health, establishes the maximum retail prices for the private market. (Organic Health Law, art. 159. Law of Generic Medicines. Ecuador. Art. 3.) The National Council is responsible for establishing price ceilings, responding to pricing requests for new medicines on the market, applying the price fixing regime for medicines in particular circumstances including emergencies, issuing instructions or resolutions for analysing, evaluating, executing and controlling the pricing policies, and verifying the controlling entities and information, requesting price information from importers or relevant legal entities; and promoting and coordinating international cooperation, technical advice and information exchange with other competent authorities; and publish a list online of the prices set for each segment of the market. (Regulations for Medication Price Setting, art. 4) In order to use the Released Price Regime (that applies to non-strategic medicines that are not subject to price control), applicants (companies) must notify the Council every 6 months of the retail price and previous monthly sales. (Regulations for Medication Price Setting, art. 26) Failure to submit this information can result in sanctions against applicants (Regulations for Medication Price Setting, art. 34). The National Agency for Sanitary Regulation, Control and Surveillance is responsible for carrying out price controls to establish compliance with the prices set by the Council. In case of a breach, the Council can apply the Direct Fixation Price Regime after several administrative steps. (Regulation Medication Price Setting, art. 36)

The National Competent Authority of Intellectual Rights is attached to the Secretary of Higher Education, Science, Technology and Innovation. The competent authority regulates, manages, and controls IPR and competition. (Organic Code of the Social Economy of Knowledge, Creativity and Innovation, art. 10) The Market Power Control Superintendency can initiate an investigation of a economic operator (other than the importer or national laboratory) that markets medicines over the ceiling price or at an increase greater than inflation, medicines that are new without fixing ceiling prices, based on untrue or falsified information provided to the Board (Regulation on Medication Price Setting, art. 21,24). Compulsory licenses may be granted (ex officio or at the request of a party) to remedy anticompetitive practices by the patent holder for reasons of public interest, emergency or national security. (Organic Code of the Social Economy of Knowledge, Creativity and Innovation, art. 315) The National Competent Authority permits non-commercial use by a government entity or contractor without prior negotiation with the patent holder, as well as third party access to undisclosed information (i.e. test data) for reasons of public interest, national emergency or extreme urgency. (Organic Code of the Social Economy of Knowledge, Creativity and Innovation, art. 314, 510) Data exclusivity is granted for five years from the date of marketing approval. (Organic Code of the Social Economy of Knowledge, Creativity and Innovation, art. 509)

Medications must be labelled with the retail price, which may not exceed the ceiling price set by the Council. (Regulation on Medication Price Setting, art. 33; Organic Law for Consumer Protection, art. 15) In addition, pharmacies and dispensers must provide consumers with access to all the retail prices at the point of sale so consumers can compare prices. (Regulation on Medication Price Setting, art. 33) Failure to comply will lead to penalties. (Regulation on Medication Price Setting, art. 33)

The National Health Council publishes the National Therapeutic Registry that includes therapeutic information of the medicines included in the National Essential Medicines List. (Law of Generic Medicines, art. 12) The Council will publish online a complete list of medicines for human use with the corresponding price. (Regulation on Medication Price Setting, art. 27)

Ghana

The Constitution of the Republic of Ghana (1992) prescribes the right to health and obligations of the government in providing citizens with quality health care. Public Health Act No. 851 (2012) prescribes the patient's right to quality basic health care irrespective of the patient's geographical location. This law also outlines the Patient Charter and the rights of the patient.

A National Health Insurance system is established by the National Health Insurance Act No. 852 of 2012. The National Health Insurance Oversight Committee advises the Board on the benefits package for the national health insurance scheme.

The National Health Insurance Authority develops a National Health Insurance Scheme Medicines List and Medicines Tariff (reimbursement price) based on the Essential Medicines List (NHI Act No. 852 of 2012, 33). The National Medicines Selection Committee (NMSC) establishes the EML with a subgroup for evidence synthesis, which is approved by the Minister of Health. The procedures and selection criteria are published with the Essential Medicines List (EML). The Medicines List and Tariff is reviewed annually (according to legislation, but every 2 years in practice) with healthcare providers, at which time medicines may be added or deleted and medicines prices may be reviewed. (NHI Act No. 852 of 2012, 33) Medicines are listed by generic name, unless it is necessary to use the brand name. (NHI Act No. 852 of 2012, 33) Any inclusion/deletions of medicines and other health technologies on the essential medicines list is based on the evidence of safety and efficacy as well as evidence from economic evaluations. Medicines considered during the review must be registered for use in the country by the national Food and Drugs Authority. Medicines listed on the EML inform procurement and reimbursement decisions within the health system. The selection of medicines takes into account the different skills of prescribers at different levels of the health care system. (National Medicines Policy, 2017).

The HTA Technical Working group is established to support the work of the NMSC with economic evaluations and also support reimbursement decisions of the NHIA. The Minister prescribes the healthcare benefits package under the National Health Insurance Scheme. (NHI Act No. 852 of 2012) The Authority must provide members with information about the benefits package, their rights, responsibilities, and complaints / dispute resolution mechanisms available under the Scheme at the point of registration. (NHI Act 852 of 2012)

The Appropriation Act 984 (2018) provides for the allocation government funds for priorities including Health from the consolidated fund and other funds during the financial year. The National Health Insurance Authority (NHIA) shall within three months after the passing of the Appropriation Act submit to Parliament for approval, the formula for the disbursement of the national health insurance fund. The disbursement formula must take into account the sustainability of the insurance scheme. (National Health Insurance Act No. 852 of 2012)

With regards to intellectual property management, granting a compulsory license in Ghana is covered under Section 13 of Ghana's Patents Act (Patents Act, 2003 (Act 657). Conditions for issuing compulsory license include public interest, national security, nutrition, health, the development of other vital sectors of the national economy, and/or the need to remedy anti-competitive practices so declared by a judicial or administrative process. Compulsory licenses are issued through coordinated action between the Ministry of Justice and Attorney General's Department; the Registrar General's Department; Ministry of Health; and Food and Drugs Authority (Regulatory agency).

Philippines

Constitutional law, medicines policy, and legislation affirm the government's duty to "endeavour to make essential goods, health, and other social services available to all people at an affordable cost," with priority for vulnerable groups and including free care to 'paupers' (i.e. 1987 Constitution art. 13, sec. 11; Philippine Medicines Policy (2011-2016) pg. 10; National Health Insurance Act revised by Republic Act No. 10606 of 2013 art. 1, sec. 1).

According to the legislation, PhilHealth Members and their dependents are entitled to minimum services including prescription drugs and biologicals in in-patient and out-patient care. (NHI Rules of 2013, sec. 10,37). However, there is currently no systematic mechanism to enforce inclusion of

medicines in outpatient care in Philhealth benefit packages. There is no system that integrates pharmacies into a system that allows them to be reimbursed by the government/Philhealth, or for patients to access medicines through drugstores without paying out-of-pocket.

The Philippine National Formulary is the basis for the selection and procurement of medicines in the public sector; it also governs the reimbursement of drugs by PhilHealth in both public and private health facilities. (Sec. 37 of Republic Act No. 7875, as amended by Republic Act No. 9241 and No. 10606). Importantly, the PhilHealth Corporation may choose not to cover expenses for health services considered cost-ineffective, as determined through a health technology assessment (Republic Act No. 10606 of 2013 art. 3 sec. 11)

The service package is reviewed annually for financial sustainability, relevance to health innovations, and quality assurance in order to increase benefits and reduce OOP. NHI Rules of 2013, sec. 37 Health services that are cost-ineffective (determined through HTA) are not financed by the PhilHealth Corporation, which may introduce additional exclusions or limitations to ensure financial sustainability. NHI Rules of 2013, sec. 38 The Department of Health is to publish annually in at least two general newspapers the generic names, corresponding brand names of all medicines available in the Philippines. Republic Act No. 9502, sec. 5

An independent price negotiation board negotiates prices with manufacturers on behalf of the Department of Health and PhilHealth. Negotiations are guided by parameters specific to new technology, innovator drugs, and single supplier products. The negotiated price is applicable to all healthcare providers under DOH. (Republic Act No. 11223 of 2018. Sec 28(b)) Maximum retail prices of medicines will be imposed on medicines by the Secretary of the Department of Health when the public interest so requires. (Republic Act No. 9502 of 2007) Medicines subject to price regulation include all medicines for the treatment of chronic illnesses and life threatening conditions, HIV-AIDS, organ transplants, and neoplasm, for the prevention of diseases (i.e. vaccines) and of pregnancy (i.e. contraception), aesthetic agents, IV fluids, medicines on the National Drug Formulary Essential Drug List, and other medicines determined from time to time. (Republic Act No. 9502 of 2007, sec 23) The Secretary may also implement competitive bidding, price-volume negotiations and other measures to influence supply, demand and expenditures on medicines. (Republic Act No. 9502 of 2007, sec 17)

The Secretary of the Department of Health holds the power to include other drugs and medicines in the list subject to price negotiation, when it is necessary in the public interest. (Republic Act no. 9502 of 2007), sec. 19) The President is empowered to impose maximum retail prices on drugs and medicines, on the recommendation of the Secretary(Republic Act no. 9502 of 2007), sec. 17).

Medicines on the Philippine National Drug Formulary Essential Drug List are subject to price regulation. (Republic Act No. 9502 of 2007, sec 23). Republic Act 9502 in 2008 has given price regulation authority to the President of the Philippines upon recommendation of the Secretary of Department of Health. The same law authorized the Secretary of Health to establish and initiate a price monitoring and regulation system for drugs, namely the Drug Price Advisory Council that established in August 2018. It is prohibited for any entity (producer, manufacturer, importer, storer, transporter, distributer, seller) to manipulate prices of any basic necessity through forming a cartel (agreement between 2+ entities to artificially and unreasonably increase or manipulate prices), hoarding (undue accumulation of any basic commodity or unreasonable limit/refusal to dispose of, sell or distribute stocks), and profiteering (sale/offering of sale of a basic necessity at a price grossly in excess of its true worth) (Republic Act No. 7581 of 1992) Unless otherwise declared, the prices of basic necessities shall automatically be frozen at their prevailing prices when the area is declare a disaster area, under emergency or martial law, or in a state of rebellion or war. (Republic Act No. 7581 of 1992, sec 6) In these situations a mandated price ceiling may also be imposed. (Republic Act No. 7581 of 1992, sec. 7)

Every manufacturer, importer, distributor, wholesaler, trade and retailer must display the retail price on medicinal products, which should not exceed the maximum retail price. From time to time manufacturers, importers, and traders shall issue a price list to wholesalers, distributors, and retailers, and the Secretary of the Department of Health indicating the retail price and maximum retail price. (Republic Act 9502, sec 26)

Patentability criteria require the invention involves an 'inventive step' that is not obvious to a person skilled in the same domain at the time of filing. (Republic Act 9502, sec 6) What constitutes an 'inventive step' with regards to pharmaceuticals is defined in legislation. (Republic Act 9502, sec 6) A government agency or third party authorised by the government may exploit the invention without the agreement of the patent owner. (Republic Act 9502, sec 8) In case full competition is not effective, the State recognizes the regulation of medicines prices by an implementing authority as one of the means to promote and ensure access to quality, affordable medicines. (republic Act 9502, sec 2) With regard to pharmaceuticals this may be in the case of a national emergency or other extreme urgency, there is public non-commercial use of the patent by the patentee without satisfactory reason, or the demand for a patented medicine is not being adequately met in the Philippines and on reasonable terms. (Republic Act 9502, sec 8) A Compulsory License may be granted by the Director General of the Intellectual Property Office in case of a national emergency or extreme urgency, where the public interest, health, or development of other vital sectors of the national economy so requires, where the patent owner's manner of exploitation is deemed anti-competitive, there is public non-commercial use of the patent by the patentee without satisfactory reason, or the demand for a patented medicine is not being adequately met in the Philippines and on reasonable terms. (Republic Act 9502, sec 10) Compulsory licenses are available for the import of a patented medicine, (local) manufacture, and export of medicines to a country with insufficient manufacturing capacity. Republic Act 9502, sec 10

South Africa

South Africa's 'transformative' post-apartheid constitution of 1996 introduced the right of everyone 'to access to healthcare services including reproductive health care' and to social security (sec. 27(1)). Under constitutional law, the State is required to take reasonable legislative and other measures to progressively realize these rights within its available resources and that no one may be refused emergency medical treatment (sec. 27(2-3)).

The National Health Insurance Act No. 61 of 2003 (amended in 2013) governs the provision of health services in the public and private sectors in accordance with available resources (art. 2). This Act provides free health services to vulnerable groups such as pregnant and lactating women and children under age 6, as well as free primary health services to the uninsured (art. 4). However, it falls short of expressing a universal right and guaranteed access to essential medicines.

The Minister, based on the recommendation of the Pricing Committee, makes regulations on the introduction of a transparent pricing system for all medicines marketed in South Africa, and appropriate wholesaler/distributor and dispensing fees. (Medicines and Related Substances Act 101 of 1965, amended 2002) Medicines may not be supplied using a bonus, rebate or other incentive scheme. (Medicines and Related Substances Act 101 of 1965, amended 2002, 18A) The Minister introduces a transparent pricing system for all medicines, including an appropriate dispensing fee charged by wholesalers/distributors and pharmacists, and which includes a single exit price (SEP) representing the manufacturers selling price, determined by the Director-General. (Medicines and Related Substances Act 101 of 1965, amended 2002, 22) Using international benchmarks for price setting is still under discussion. The logistics fee, however, is determined in agreement by the logistics provider and manufacturer/importer; it must be below the maximum logistics fee supplied to the Minister as part of the SEP. (Medicines and Related Substances Act 101 of 1965, amended 2002, 22) The SEP may be increased only once a year. Pharmacists, wholesalers and distributors shall not sell a medicine above the SEP. (Medicines and Related Substances Act 101 of 1965, amended 2002, 22) The SEP must be clearly labelled on the medicines package sold to the user. (Regulations relating to a transparent pricing system for medicines 2004, 4) The Director-General may publish or communicate, or require manufacturers, importers, wholesalers, distributors, or pharmacists to communicate about a Schedule of medicines in order to inform the public about the SEP, therapeutic value and risks relative to the price, availability, applicable pricing system, supply chain, and fees charged by wholesalers, distributors, retailers, and other sellers. (Regulations relating to a transparent pricing system for medicines 2004, 21 (1-2)).

A dominant firm is prohibited from charging an excessive price to the detriment of consumers, refusing to give a competitor access to an essential facility when it is economically feasible to do so, engage in an exclusionary act if it has a predominant anti-competitive effect, refusing to supply scarce goods to a competitor or customer when economically feasible to do so, buying up scarce supply of intermediate goods required by a competitor, and engaging in predatory pricing or margin squeeze. (Competition Amendment Act 2019, 8)

The proposed National Health Insurance Act foresees the following: The Benefits Advisory Committee must determine and review the health care service benefits reimbursed at each level of care, as well as cost-effective treatment guidelines that consider the emergence of new technologies. (National Health Insurance Act proposed) The precursor to the Benefits Advisory Committee, the Ministerial Advisory Committee on Health Care Benefits for National Health Insurance, will advise the Minister on priority setting to inform decisions about the benefits to be covered. (National Health Insurance Act proposed) Under this Act users have a right to information relating to the health care service benefits available to them and information on the funding of healthcare services. (NHI Act proposed, 6 (b,c,n)). If the Health Insurance Fund refuses to fund a service, then it must notify the user and provide the person with a reasonable opportunity to make representations and provide adequate reasons for the decision to refuse the service to the user. (National Health Insurance Act proposed, 7(5)) Treatments will not be funded under this Act if no medical necessity exists for it, no cost-effective intervention exists (determined by HTA), or the product or treatment is not included in the Formulary, unless a complementary list has been approved by the Minister. (NHIA proposed, 4)

The Minister may issue the following decisions for the supply of more affordable medicines: determine the extent of patent rights vis-a-vis medicines already marketed by the owner of the patent. (Medicines and Related Substances Act No. 101 of 1965, 2002. Sec. 15C)

Ukraine

Access to medicines is recognized as an essential part of right to healthcare in Ukraine. Everyone has a right to medical care (including access to medicines): "States ensure the availability of essential medicines, … as well as… benefits and guarantees for certain groups and categories of citizens.", art.3. Adopted in 2017, the Law on State Financial Guarantees of Health Care Services to the Population more specifically defined issues concerning access to medicines. The program of medical guarantees, basically "national package of health services", - inter alia, determines a list and scope of medicines, full payment for which the state guarantees to patients at the expense of the State Budget.

There are two mechanisms that guarantee access to medicines:

(1) Benefits and guarantees for certain groups and categories of people who may receive medicines for free or on a preferential basis. Among them are persons with disabilities (some categories); kids before 3 y.o.; children with disabilities; combatants; veterans of state special agencies (military service, law enforcement, etc); pensioners, receiving the minimum pension and others.

(2) The affordable medicine program, which was implemented in 2017. This program provides full or partial reimbursement of essential medicines for treatment of cardiovascular diseases, asthma and diabetes mellitus type 2. Expanding the program coverage is one of the future activities.

The Expert Committee for the Selection and Use of Essential Medicines is a permanent advisory body established by the Ministry of Health to assess the comparative effectiveness, safety, and economic feasibility of medicines considered for inclusion on the National List of Essential Medicines. (Position on the Expert Committee 2016, 2017. Order no. 84, 2016, 2016) Economic feasibility considers the cost of the medicines and the total cost to treat the population (Order no. 84 2016, 2017) Selected essential medicines are those to meet the priority needs for treatments at the expense of public budgets. (Position on the Expert Committee 2016, 2017. Ukraine) The structure and content of the National List is based on the recommendations by WHO. The National List includes both registered and unregistered medicines in the Ukraine. (Order No 84, 2016, 2017) Amendments to the National List can be initiated by the Committee, individuals and legal entities. Criteria for making amendments include the disease burden, comparative effectiveness, safety, and economic feasibility, budget impact, pharmacokinetic properties, and WHO's

recommendation. (Order No 84, 2016, 2017) Information on the initiator of new applications and their contents, as well as the Expert Committee's own report on each application shall be published on the Expert Committee's website. (Order No 84, 2016, 2017, 4,9,10) The State guarantees funding for medicines and medical services included in the program of medical guarantees. (Law No. 2168-VIII, 2017, 2020) Medicines included in the Register of Medicinal Products Subject to Reimbursement are subject to price regulation. (Decree No. 135 of 2019) The amount of reimbursement is calculated using internal and external reference pricing, marginal wholesale pricing, and minimum wholesale prices. (Decree No. 135 of 2019) In 2017 a pilot project was launched to introduce state regulation of insulin prices. (Decree No. 73 2014, 2017)

Medicines in the National List of Essential Medicines are procured by health care institutions that are fully or partially financed by state and local budgets. (Decree no. 333 of 2009, 2017) Medicines are purchased on the principle of maximum economy and efficiency. (Decree no. 333 of 2009, 2017) These rules do not apply to medicines manufactured in pharmacies, or medicines procured through specialised agreements or procurement organizations. (Decree no. 333 of 2009, 2017) Since 2015, public procurement of medicines and medical devices has been carried out through specialized international organizations such as the UN Development Program, UNISEF, and Crown Agents. All information about procurement contracts, medicines, public funds which have been spent are published in report by the Ministry of Health of Ukraine. The State owned enterprise 'Medical Procurements of Ukraine' had started to operate in 2019. In the future this enterprise will organize and conduct public procurement of medicines using State budget funds. At the moment, legal regulations about its functioning is still under development.

The National Health Service of Ukraine (NHSU) reviews proposal for including medicinal products in the register together with the amount of reimbursement, surcharge, and price for the medicine. (Decree No. 135, 2019) The National Health Service of Ukraine calculates the prices of medicines, the amount of reimbursement and the amount of surcharges. (Decree no. 135, 2019.) Tariffs for the provision (reimbursement) of medicines under the medical guarantee program are established. (Law No. 2168-VIII, 2017, 2020) Reimbursement for the costs of medicines is carried out by the Authorized Body. (Law No. 2168-VIII, 2017, 2020)

The Central Executive Body advises on the issuing of patents for medical products. (Law No. 123.96 BP, 1996, 2020) It is also obliged to provide free access to all results of preclinical studies and clinical trials on its official website, if such information is publicly accessible (Law No. 123.96 BP, 1996, 2020, art. 9(11)) Data exclusivity provisions apply for five years after the market authorisation of the first product. This can be extended by six years if in the first three years after market approval the product was marketed for one or more indications considered to be of special advantage over existing ones. Law No. 123.96 BP, 1996, 2020, art.9(12-13)) The Cabinet of Ministers may allow a designated entity to use a registered patented invention without consent of the owner (utility model). Law No. 3687.12 BP, 1993, 2020, art. 30).

Patients have the right to receive information from public authorities or health providers regarding the program of medical guarantees, as well as the information about one's own state of health and related risk factors. (Law No. 2801-XII 1992, 2020; Law No. 2168-VIII, 2017, 2020) There is no legislation that specifies patients have a right to receive information about the cost/prices. Health providers are obliged to inform patients about the medicines they can receive from this provider under the medical guarantee program. Law No. 2168-VII 2017, 2020 Pharmaceutical workers may not conceal information from consumers such as the presence of medicines at a lower price otherwise they may be liable under the law (Law No. 2801-XII 1992, 2020, art. 78-I)

At least once a month the NHSU publishes on its website the trade names of medicinal products subject to reimbursement, the amount of reimbursement and surcharge (if partially reimbursed), and the retail price per package. (Decree no. 135 of 2019, 11(6-7)) Ministry of Health of Ukraine publishes on its official website the INN, trade name, form of release and dose of product for which a controlled access agreement (managed access agreement) is concluded. Law No. 2801-XII 1992, 2020.

© 2020 by the authors. Submitted for possible open access publication under the terms and conditions of the Creative Commons Attribution (CC BY) license (http://creativecommons.org/licenses/by/4.0/).
